# The glyceraldehyde-3-phosphate dehydrogenase GapDH of *Corynebacterium diphtheriae* is redox-controlled by protein *S*-mycothiolation under oxidative stress

**DOI:** 10.1038/s41598-017-05206-2

**Published:** 2017-07-10

**Authors:** Melanie Hillion, Marcel Imber, Brandán Pedre, Jörg Bernhardt, Malek Saleh, Vu Van Loi, Sandra Maaß, Dörte Becher, Leonardo Astolfi Rosado, Lorenz Adrian, Christoph Weise, Rüdiger Hell, Markus Wirtz, Joris Messens, Haike Antelmann

**Affiliations:** 1 Institute for Biology-Microbiology, Freie Universität Berlin, D-14195 Berlin, Germany; 2 Center for Structural Biology, VIB, B-1050 Brussels, Belgium; 3 Brussels Center for Redox Biology, B-1050 Brussels, Belgium; 4 Structural Biology Brussels, Vrije Universiteit Brussel, B-1050 Brussels, Belgium; 5 Institute for Microbiology, Ernst-Moritz-Arndt-University of Greifswald, D-17487 Greifswald, Germany; 6 Department Isotope Biogeochemistry, Helmholtz Centre for Environmental Research-UFZ, Leipzig, Germany; 7 Institute for Chemistry and Biochemistry, Freie Universität Berlin, D-14195 Berlin, Germany; 8 Plant Molecular Biology, Centre for Organismal Studies Heidelberg, University of Heidelberg, Heidelberg, Germany

## Abstract

Mycothiol (MSH) is the major low molecular weight (LMW) thiol in Actinomycetes and functions in post-translational thiol-modification by protein *S*-mycothiolation as emerging thiol-protection and redox-regulatory mechanism. Here, we have used shotgun-proteomics to identify 26 *S*-mycothiolated proteins in the pathogen *Corynebacterium diphtheriae* DSM43989 under hypochlorite stress that are involved in energy metabolism, amino acid and nucleotide biosynthesis, antioxidant functions and translation. The glyceraldehyde-3-phosphate dehydrogenase (GapDH) represents the most abundant *S*-mycothiolated protein that was modified at its active site Cys153 *in vivo*. Exposure of purified GapDH to H_2_O_2_ and NaOCl resulted in irreversible inactivation due to overoxidation of the active site *in vitro*. Treatment of GapDH with H_2_O_2_ or NaOCl in the presence of MSH resulted in *S*-mycothiolation and reversible GapDH inactivation *in vitro* which was faster compared to the overoxidation pathway. Reactivation of *S*-mycothiolated GapDH could be catalyzed by both, the Trx and the Mrx1 pathways *in vitro*, but demycothiolation by Mrx1 was faster compared to Trx. In summary, we show here that *S*-mycothiolation can function in redox-regulation and protection of the GapDH active site against overoxidation in *C*. *diphtheriae* which can be reversed by both, the Mrx1 and Trx pathways.

## Introduction

Bacteria are exposed to various redox-active compounds, such as reactive oxygen species (ROS) in their natural habitat or during infections and are equipped with specific protection mechanisms^1^. To cope with ROS, bacteria use different antioxidant enzymes, such as catalases, peroxiredoxins, superoxide dismutase and low molecular weight (LMW) thiols to maintain the reduced state of the cytoplasm and to survive oxidative stress^2–4^. Gram-negative bacteria utilize glutathione (GSH) as their major LMW thiol, but GSH is absent in most Gram-positive bacteria. Instead, the Actinomycetes that include streptomycetes, corynebacteria and mycobacteria produce mycothiol (MSH) as their major LMW thiol^5^. MSH functions in detoxification of various redox-active compounds, including ROS, electrophiles and antibiotics in all Actinomycetes^6–8^. Apart from its detoxification functions, MSH is also involved in post-translational thiol-modification and forms mixed disulfides with protein thiols under hypochlorite stress^9, 10^. Protein *S*-mycothiolation is an emerging thiol-protection and redox-regulatory mechanism in Actinomycetes. In *Corynebacterium glutamicum*, we identified 25 *S*-mycothiolated proteins using shotgun LC-MS/MS analysis^10^. These include conserved targets for *S*-thiolation across different Gram-positive bacteria, such as the thiol-peroxidase Tpx, the inosine monophosphate (IMP) dehydrogenase GuaB and ribosomal proteins^10, 11^. In *Mycobacterium smegmatis*, protein *S*-mycothiolation was more abundant with 58 identified proteins, which correlates with the 20-fold higher MSH content in mycobacteria compared to corynebacteria^9^.

The redox-regulatory mechanisms of *S*-mycothiolated proteins have been studied thus far for several antioxidant enzymes, such as thiol peroxidases (Tpx, Mpx, AhpE) and methionine sulfoxide reductases (MsrA)^10, 12–15^. Moreover, Tpx has been shown to function as a peroxidase and as oligomeric chaperone in response to different levels of H_2_O_2_^15^. Regeneration of peroxidase and methionine sulfoxide reductase activities requires both the mycoredoxin (Mrx1) and thioredoxin pathways *in vitro* ^10, 12, 13, 16, 17^. Apart from its redox-regulatory role for antioxidant enzymes, MSH also functions in thiol-protection of the methionine synthase MetE by protein *S-*mycothiolation under acid stress conditions^18^.

In this work, we have used shotgun proteomics to identify 26 *S*-mycothiolated proteins in the pathogen *Corynebacterium diphtheriae*. As major redox-controlled metabolic enzyme, the glycolytic glyceraldehyde-3-phosphate dehydrogenase DIP1310 (GapDH) was *S*-mycothiolated under NaOCl stress at the active site Cys in *C*. *diphtheriae in vivo*. GapDH is a conserved target for redox-regulation and post-translational thiol-modifications including S-glutathionylations across all domains of life^19, 20^. In *Staphylococcus aureus*, the glycolytic GapDH was recently shown as major target for *S*-bacillithiolation which contributes with 4% to the total Cys proteome^21^. GapDH uses the active site Cys for the nucleophilic attack at the aldehyde group of glyceraldehyde-3-phosphate (G3P) to catalyze its phosphorylation to 1,3-bisphosphoglycerate, generating NADH in this process^20^. The relatively high reactivity of the active site thiolate towards H_2_O_2_ depends on the stabilization of the transition state and a dedicated proton relay mechanism that promotes leaving group departure^20, 22^. *S*-glutathionylation of GapDH from the plant *Arabidopsis thaliana* resulted in enzyme inactivation which could be faster regenerated by glutaredoxins compared to thioredoxins^23^. Here, we studied the redox-regulation of GapDH of *C*. *diphtheriae* in response to oxidative stress by protein *S*-mycothiolation *in vitro*. We show that *S*-mycothiolation functions in redox regulation and efficiently protects the active site against overoxidation by H_2_O_2_ and NaOCl which can be reversed by both, the Mrx1 and Trx pathways. Thus, striking similarities exist in the redox-control mechanisms of GapDH homologs from prokaryotic and eukaryotic organisms that involve protein *S*-thiolations using different thiol-redox systems for recycling, and as such for controlling central glycolytic activities.

## Results

### Identification of 26 *S*-mycothiolated proteins *in C*. *diphtheriae* under NaOCl stress using shotgun LC-MS/MS analysis

**Figure 1:**
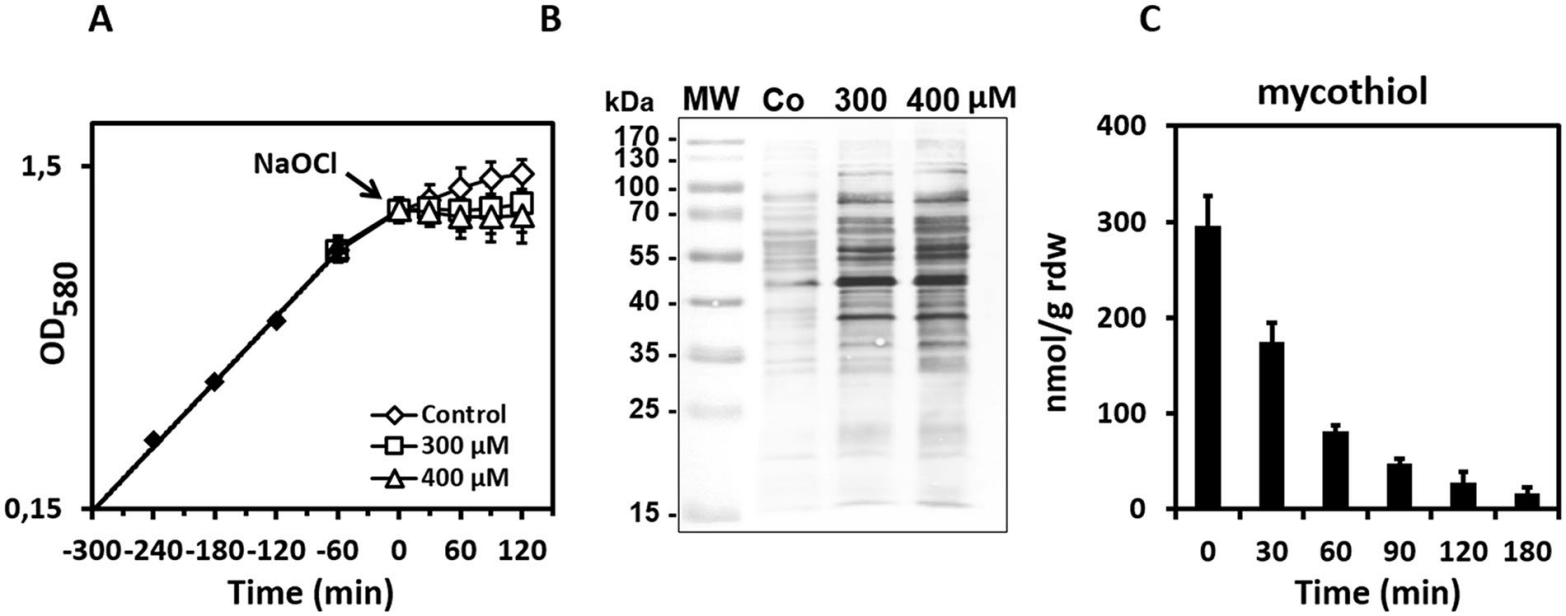
Protein *S*-mycothiolation pattern and MSH depletion in *C*. *diphtheriae* under NaOCl stress. *C*. *diphtheriae* was grown in HIB medium to an OD_580_ of 0.75–0.8, transferred to BMM and treated with 300 and 400 µM NaOCl which resulted in growth delay **(A)** and strongly increased protein *S*-mycothiolation as revealed by non-reducing MSH-specific Western blots **(B)**. The level of reduced MSH was 0.3 µmol/g rdw in the control and strongly depleted under NaOCl stress in the thiol-metabolome indicating that MSH is used for protein *S-*mycothiolation **(C)**. All data represent mean values of three independent biological replicates and the error bars given were calculated as standard error of the mean (SEM).

The role of protein *S*-mycothiolation in thiol-protection and redox regulation has been studied previously in *C*. *glutamicum* ^10^ and *M*. *smegmatis* ^9^. In this study, we were interested to identify the targets for protein *S*-mycothiolation in the pathogen *C*. *diphtheriae* under NaOCl stress. Cells of *C*. *diphtheriae* DSM43989 were grown in heart-infusion broth (HIB) and transferred at an OD_580_ of 0.8 into a minimal medium (BMM) for NaOCl stress exposure to avoid the quenching of NaOCl by the rich HIB medium. Treatment of cells with 300 µM and 400 µM NaOCl resulted in a delay of growth with slow recovery after overnight growth **(**Fig. 1A **)**. Using MSH-specific non-reducing Western blots, a strongly increased protein *S*-mycothiolation pattern could be detected after 30 min of 300–400 µM NaOCl stress **(**Fig. 1B **)**. We further analysed the MSH level in *C*. *diphtheriae* under NaOCl stress using thiol-metabolomics. The MSH level was determined as 0.3 ± 0.03 µmol/g raw dry weight (rdw) under control conditions which decreased 4-fold after 30 min of NaOCl treatment **(**Fig. 1C **)**. Thus, the depletion of MSH correlates with increased protein *S*-mycothiolation under NaOCl stress. This confirms our previous results in *M*. *smegmatis* where strong MSH depletion was also observed under NaOCl stress^9^.

**Figure 2:**
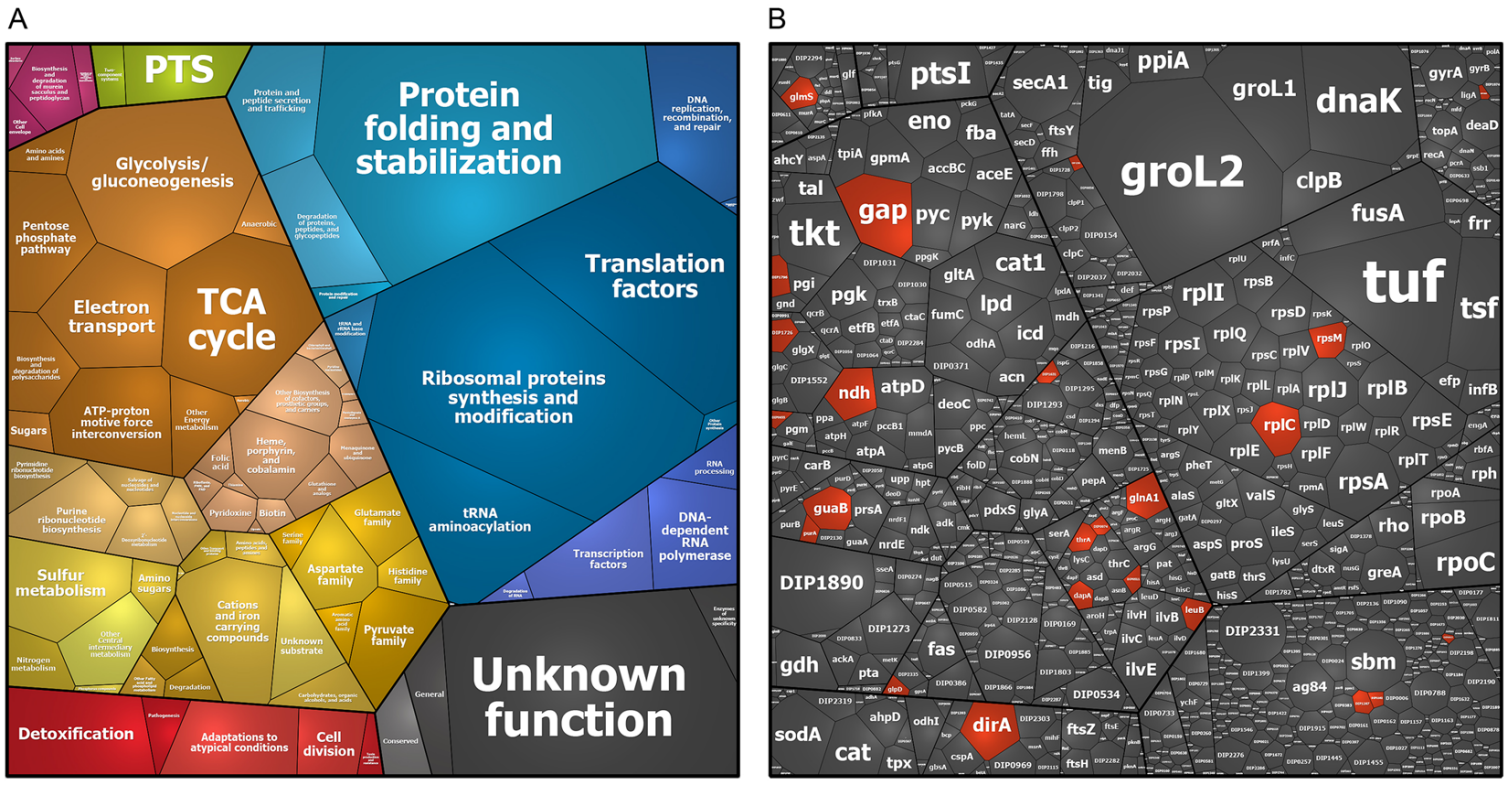
Voronoi treemaps show total protein abundance and 26 *S-*mycothiolated proteins identified in *C*. *diphtheriae* under NaOCl stress using shotgun LC-MS/MS analysis. (**A**) The treemap legend shows the classification of the *C*. *diphtheriae* proteome into functional categories as revealed by TIGRfam annotations. (**B**) The spectral protein abundance determines the cell size of each protein identified in the total proteome (Table S3). The 26 identified *S-*mycothiolated proteins under NaOCl stress are red-colored in the proteome treemap. The protein abundance treemap indicates that Gap, DirA (AhpC), Ndh and GuaB belong to the most abundant *S*-mycothiolated proteins in the total proteome.

Using LTQ-Orbitrap LC-MS/MS analysis, we identified 26 *S*-mycothiolated proteins in *C*. *diphtheriae* in NaOCl-treated cells based on the 484 Da mass increase of MSH at cysteine residues **(**Tables 1, S1 and S2 **)**. These *S*-mycothiolated proteins are displayed in a Voronoi treemap where the spectral protein abundance determines the cell size of each protein that is present in the proteome and the *S*-mycothiolated proteins are marked in red **(**Fig. 2 **)**. The 26 *S*-mycothiolated proteins of *C*. *diphtheriae* include only 5 conserved targets for *S*-thiolation, such as the peroxiredoxin AhpC, the ribosomal proteins RplC and RpsM, the glycolytic enzyme glyceraldehyde-3-phosphate dehydrogenase (GapDH) and the IMP dehydrogenase GuaB **(**Tables 1, S1 and S2 **)**. The ribose 5-phosphate isomerase DIP1796 was identified as *S*-mycothiolated in *C*. *diphtheriae* which functions in the pentose phosphate pathway and was previously found *S*-glutathionylated in the photosynthetic organism *Chlamydomonas reinhardtii* ^24^. In *Leishmania*, this enzyme is essential for replication of the intracellular form of the parasite, and in *Trypanosoma brucei* the knockout mutant has a reduced infectivity in mice^25^. Other *S*-mycothiolated proteins are involved in energy metabolism (Ndh, GlpD, DIP1726), amino acid biosynthesis pathways (ThrA, LeuB, DapA, GlnA), purine biosynthesis (PurA), iron sulfur cluster biosynthesis (DIP1631) and cell wall biosynthesis (GlmS). The NADH dehydrogenase (Ndh) is an abundant enzyme that plays a role in the respiratory chain. *S*-mycothiolation of Ndh was found at the non-conserved Cys159. Some *S*-mycothiolated proteins are Cys-rich proteins including the glutamine synthetase GlnA1, the 4-alpha-glucanotransferase MalQ (DIP1726), and PurA, which possess 4 to 8 Cys residues. GlnA1 catalyzes the condensation of glutamate and ammonia to form glutamine and plays a major role in the survival of *Mycobacterium tuberculosis* under infection inside macrophages^26^. In conclusion, the identified *S*-mycothiolated proteins are mainly involved in cellular metabolism, and share as main and conserved targets for *S*-thiolations: GapDH, GuaB, AhpC and the ribosomal proteins RplC and RpsM.

**Table 1:** Identification of 26 *S*-mycothiolated proteins in *C. diptheriae* DSM43989 using shotgun LC-MS/MS analysis after exposure to 400 μM NaOCl for 30 min.

Protein	Locus Tag	Function	Cys-SSM	Cys-SSM peptide sequence	Ortholog and conservation* of Cys with -SSM in *Mtb*
**Antioxidant enzymes**					
DirA (AhpC)	DIP1420	2-Cys peroxiredoxin	Cys61* ^**#**^ **active site**	(K)DFTFVC_61_PTEIAAFGK(L)	Rv2428*
**Protein synthesis**					
RplC	DIP0473	50S ribosomal protein L3	Cys154* ^**#**^	(R)VGGIGAC_154_ATPGR(V)	Rv0701*
RpsM	DIP0546	30S ribosomal protein S13	Cys86* ^**#**^	(K)IEIGC_86_YQGLR(H)	Rv3460c*
Pth	DIP0897	Peptidyl-tRNA hydrolase	Cys49	(K)ASGAVIEVGGC_49_R(V)	Rv1014c
DIP1398	DIP1398	RNA methyltransferase	Cys376* nucleophile	(R)AIAQSGPQAAIHIGC_376_DPATFAR(D)	Rv2689c*
**Energy metabolism**					
DIP1726	DIP1726	Putative glucanotransferase	Cys45	(R)SLGVC_45_FGNEDEPATDHEPLTGPMPSEDQIR(Y)	Rv1781c
Gap	DIP1310	Glyceraldehyde 3-phosphate DH	Cys153* ^**#**^ **active site**	(K)HNIISNASC_153_TTNCLAPMAK(V)	Rv1436*
DIP1796	DIP1796	Putative ribose/galactose isomerase	Cys143	(R)RIDILC_143_EYER(T)	Rv2465c
DIP0655	DIP0655	Putative ribokinase	Cys171	(R)GTVVVNLAPVIDVDRDC_171_LLR(A)	—
GlpD	DIP2237	Putative glycerol-3-phosphate DH	Cys10	(K)SHC_10_TFNPDYYQDVWQR(F)	Rv2249c
Ndh	DIP1217	NADH dehydrogenase	Cys159	(R)AEmC_159_EDPKER(E)	Rv1854c
**Biosynthesis of amino acids**					
ThrA	DIP1036	Homoserine dehydrogenase	Cys243	(R)VTYADVYC_243_EGISK(I)	Rv1294
DIP0511	DIP0511	4-hydroxy-tetrahydrodipicolinate synthase	Cys141	(R)AVAAATSLPVIAYDIPVC_141_VHTK(L)	—
DapA	DIP1464	4-hydroxy-tetrahydrodipicolinate synthase	Cys161	(R)SVVPIAPDTLC_161_R(L)	Rv2753c
DIP0974	DIP0974	Putative aminotransferase	Cys138	(R)C_138_DAPHELPNDDIDLVFINSPSNPTGR(V)	Rv1178
GlnA1	DIP1644	Glutamine synthetase	Cys220	(R)QHPEC_220_GTGSQQEINYR(F)	—
LeuB	DIP1105	3-isopropylmalate dehydrogenase	Cys130	(R)EGTEGLYC_130_GNGGTLR(E)	Rv2995c
**Biosynthesis of nucleotides**					
DIP1631	DIP1631	Uncharacterized protein	Cys43*	(R)IAVQPGGC_43_SGLR(Y)	Rv2204c*
GuaB	DIP0580	Inosine-5′-monophosphate DH	Cys317* ^**#**^ **active site**	(K)VGIGPGSIC_317_TTR(V)	Rv3410c*
PurA	DIP2063	Adenylosuccinate synthetase	Cys423	(R)DQTIVC_423_HDVMEA(-)	Rv0357c
**Other functions**					
DIP0913	DIP0913	Uncharacterized protein	Cys22	(K)ERPTAGPQLYPVTC_22_EAVVSAIR(A)	—
DIP1026	DIP1026	Conserved ATP-binding protein	Cys75	(R)IC_75_LEADLGPVR(F)	Rv1278
DIP1102	DIP1102	Putative uncharacterized protein	Cys441	(R)LLSAC_441_PESGLYK(G)	—
DIP1250	DIP1250	M18 family aminopeptidase	Cys401*	(K)AGSSHQVFVGNNSVPC_401_GSTIGPITATR(L)	Rv0800*
DIP1287	DIP1287	UPF0210 protein DIP1287	Cys324	(K)GGMMAC_324_SR(V)	—
GlmS	DIP1700	Glutamine-fructose-6-P aminotransferase	Cys74	(K)VQALEQELETSPMPQSC_74_LGIGHTR(W)	Rv3436c

The *S*-mycothiolated proteins were identified using shotgun LC-MS/MS analysis and the Scaffold proteome software based on the mass increase of 484 Da (for -SSM) at Cys peptides. The table lists the Uniprot-accession number, protein name, conservation of the protein and the *S*-mycothiolated Cys residue in *M*. *tuberculosis (Mtb)* and the Cys-SSM peptide sequence. Conserved Cys residues are indicated with (*) and are shown in bold-face. Cys residues that were previously identified *S*-mycothiolated or *S*-bacillithiolated in *C*. *glutamicum*, *M*. *smegmatis* or *S*. *aureus* are indicated with (#).

### Contribution of ***S***-mycothiolated proteins to the total Cys proteome

**Figure 3:**
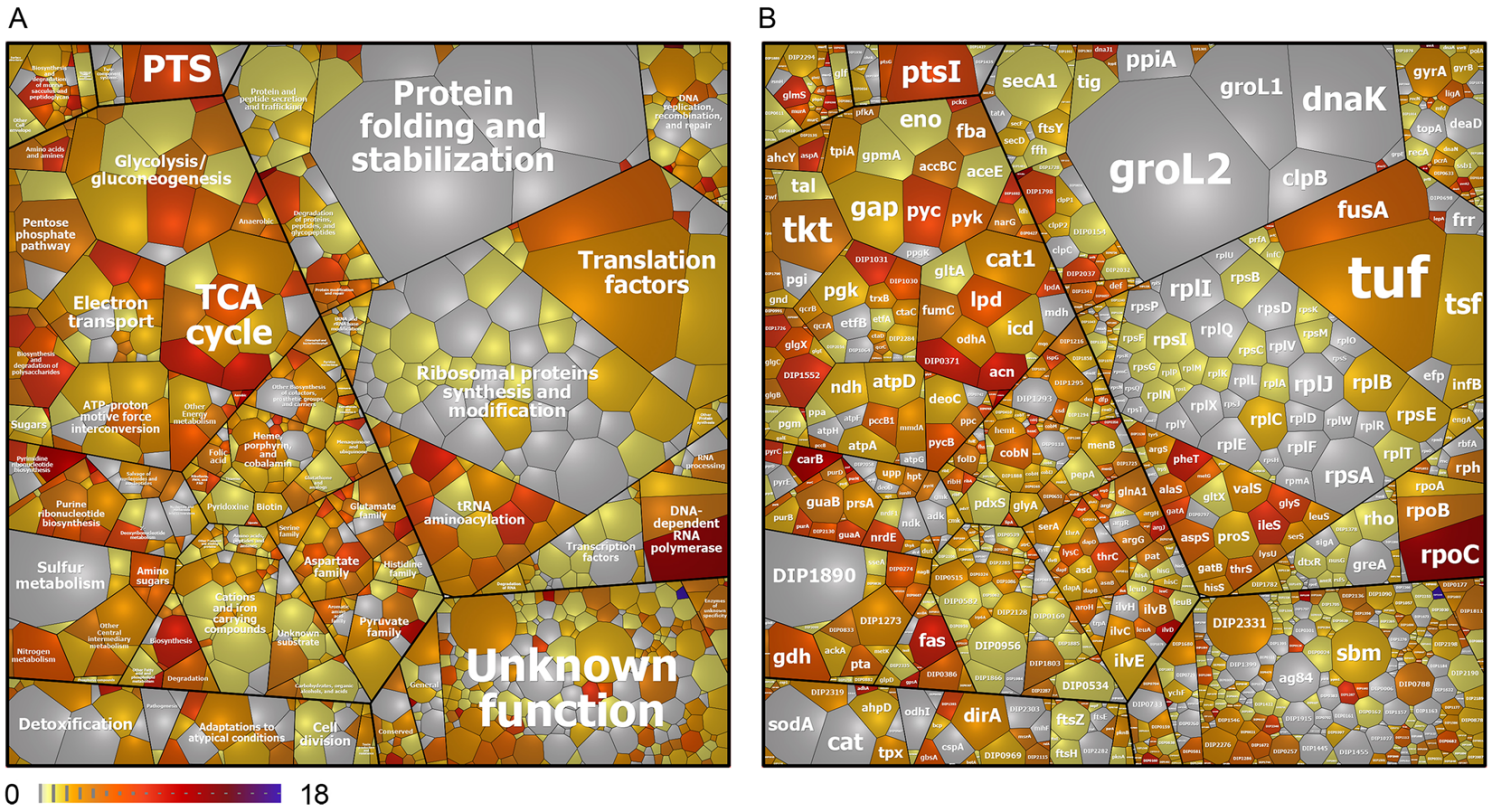
The total Cys abundance treemap of *C*. *diphtheriae* with proteins color-coded according to their number of Cys residues. **(A)** The treemap legend shows the functional classification of 1030 proteins detected in the proteome of *C*. *diphtheriae* as revealed by their TIGRfam annotations. **(B)** The spectral protein abundance determines the cell size of each protein identified in the total proteome **(**Table S3 **)**. The 805 Cys proteins were color-coded using a yellow-red color gradient based on their numbers of Cys residues. Non-Cys proteins are displayed in grey. The Cys abundance treemap visualizes that *C*. *diphtheriae* contains many Cys-rich proteins with >4 Cys residues in the proteome. The most abundant *S*-mycothiolated proteins Gap, DirA (AhpC), Ndh and GuaB contribute with 0.4–0.8% to the total Cys proteome. The values of calculated Cys abundances are shown in Table S4.

 It was interesting to note that GapDH was *S*-mycothiolated at the active site Cys153 in *C*. *diphtheriae*. Previously, we found that GapDH is the major target for *S*-bacillithiolation in *S*. *aureus* contributing with 4% Cys abundance to the total Cys proteome^21^. Thus, we calculated the percentages of Cys contributions of GapDH and other *S*-mycothiolated proteins to the total Cys proteome in *C*. *diphtheriae* (Figs 3 and S1, Table S4). In total, 2266 proteins are encoded in the genome of *C*. *diphtheriae* DSM43989 that include 1847 Cys proteins with 6156 Cys residues. The theoretical Cys content in the proteome of *C*. *diphtheriae* is 0.85% confirming that Cys is the rarest amino acid in the *C*. *diphtheriae* proteome (Figure S1). Next, we calculated the percentages of Cys abundances of all Cys proteins expressed in the proteome based on their spectral counts that are multiplied by the numbers of Cys residues. The spectral counts of the 1030 expressed proteins are visualized in a Cys proteome treemap including 805 Cys proteins (Fig. 3, Tables S3 and S4). The cell size indicates the spectral protein abundance and the color-code denotes the Cys content. About 395 Cys proteins contain only 1–2 Cys residues while the remaining 410 proteins have 3 or more Cys residues. These include 11 proteins with more than 10 Cys residues and the FeS-cluster oxidoreductase DIP2133 was identified as the most Cys-rich protein with 18 Cys residues. Of note, 83 Cys proteins were found to contribute to 60% of the total Cys abundances in the proteome including 55 Cys-rich proteins with more than 3 Cys residues (Figure S1). The RNA polymerase subunit beta’ (RpoC) and two translation elongation factors (Tuf and FusA) account for 2.5–4.5% of the total Cys abundance in the proteome. Furthermore, the Cys abundance treemap also visualizes that many ribosomal proteins and abundant chaperones and proteases (GroES, GroL1, GroL2, DnaK and ClpB) are devoid of Cys residues (Fig. 3).

Of the 26 *S*-mycothiolated proteins, 24 proteins were quantified based on their total spectral counts (Tables S3 and S4). Eleven *S*-mycothiolated proteins were found to contribute with 0.2–0.75% to the total Cys abundance, including the glycolytic GapDH on the top with 0.75%. Thus, in *C*. *diphtheriae*, GapDH is also the most abundant target for *S*-mycothiolation in comparison to all other identified *S*-mycothiolated proteins. Apart from GapDH, the AhpC homolog DirA, the IMP dehydrogenase GuaB, the glucanotransferase MalQ (DIP1726) and the glutamine synthetase GlnA1 contributed with 0.4–0.6% to the total Cys abundance in the proteome (Fig. 3; Table S4). As noted already, many *S*-mycothiolated proteins are Cys-rich proteins with more than 4 Cys residues which might explain why they are susceptible to *S*-mycothiolation under NaOCl stress. In conclusion, the comparison of the *S*-mycothiolated proteins with their Cys abundances in the total Cys proteome indicates that GapDH makes a major contribution to the *S*-mycothiolome in *C*. *diphtheriae* under NaOCl stress.

### GapDH is reversibly inhibited and protected against overoxidation by *S*-mycothiolation under H_2_O_2_ and NaOCl stress *in vitro*

GapDH was identified as *S*-mycothiolated at its active site Cys153 that is highly sensitive to oxidation by H_2_O_2_ and located in a conserved C_153_TTNC_157_ motif present in prokaryotic and eukaryotic GapDH homologs **(**Figure S2 **)**. Under peroxide stress, the active site Cys is initially oxidized to a sulfenic acid that reacts further with LMW thiols, such as GSH, leading to *S*-glutathionylation^22, 27^. In the absence of thiol-redox systems or adjacent thiols, Cys-SOH can react further to irreversible oxidation forms, such as sulfinic or sulfonic acids^1, 28^. *S*-glutathionylation functions in redox control and protects catalytic and vulnerable Cys residues against overoxidation^22, 29–31^.

**Figure 4:**
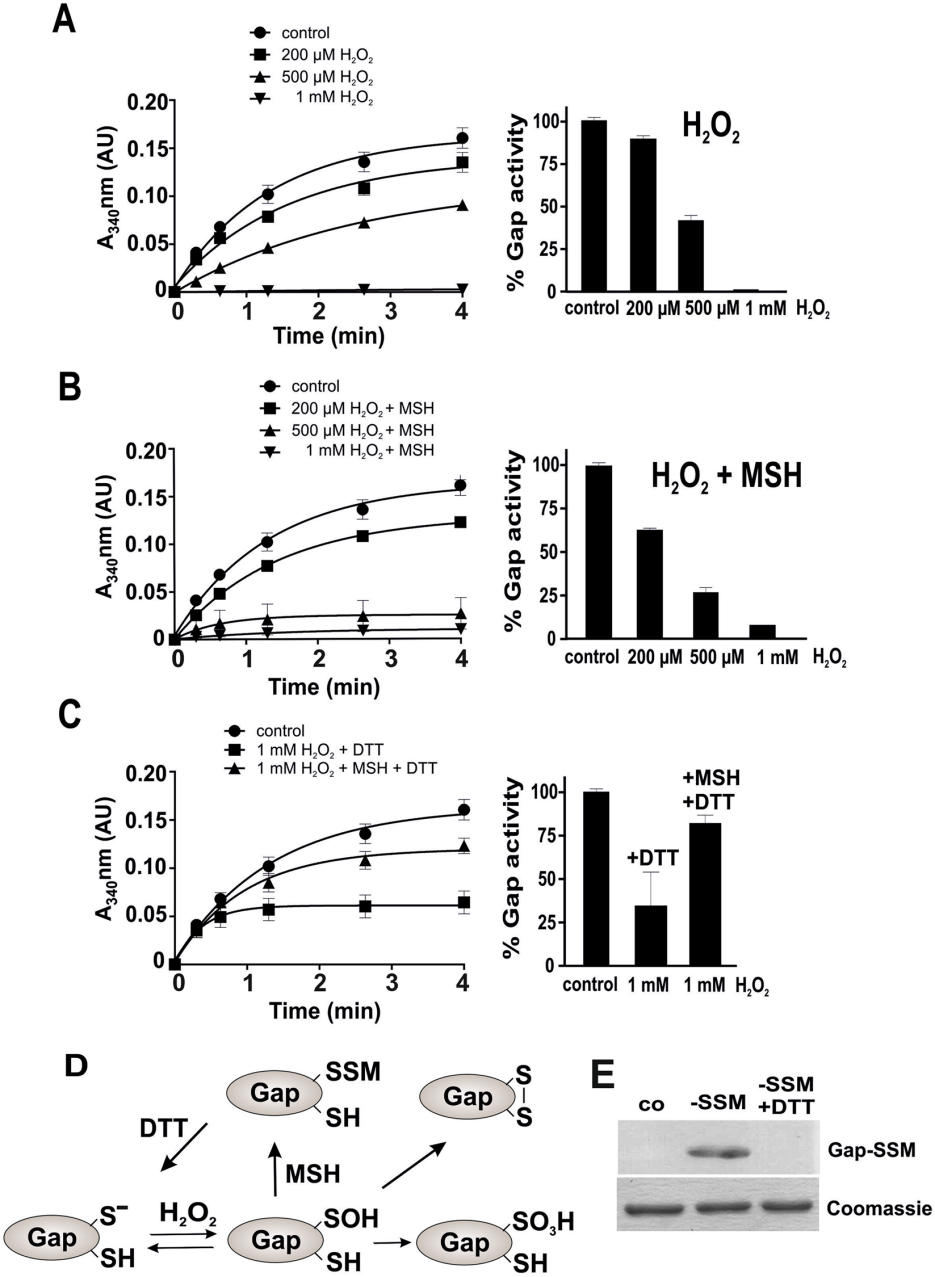
*S*-mycothiolation protects GapDH against overoxidation under H_2_O_2_ stress *in vitro*. **(A**,**B)** The NAD^+^-dependent GapDH activity was determined in a spectrophotometric assay by monitoring NADH generation during G3P oxidation at 340 nm. Inactivation of GapDH activity was performed using 200 µM, 500 µM and 1 mM H_2_O_2_ **(A)** in the absence and **(B)** in the presence of 1 mM MSH. **(A**,**C)** GapDH is 65% irreversibly inactivated with 1 mM H_2_O_2_ alone due to overoxidation of the active site Cys. **(B**,**C)** GapDH activity is reversibly inhibited due to S-mycothiolation with 1 mM H_2_O_2_ and MSH and could be reactivated by 10 mM DTT. **(E)** Non-reducing MSH specific Western blot analysis confirmed the *S*-mycothiolation of GapDH under H_2_O_2_ and MSH treatment and its reduction by DTT. **(D)** These results suggest that the GapDH active site Cys forms a sulfenic acid that reacts further to form Cys sulfonic acid and intramolecular disulfides in the presence of 1 mM H_2_O_2_ alone. GapDH is protected against this irreversible overoxidation by S-mycothiolation of the active site Cys in the presence of MSH and H_2_O_2_. All data represent mean values of three independent replicate experiments and the error bars given were calculated as standard error of the mean (SEM).

We were interested to investigate if *S*-mycothiolation controls GapDH activity and functions in thiol-protection against overoxidation *in vitro*. The His-tagged enzyme was cloned in *Escherichia* *coli*, purified and subjected to GapDH activity assays after exposure to H_2_O_2_ and NaOCl in the absence and presence of MSH *in vitro*. The inhibition of glycolytic GapDH activity by H_2_O_2_ and NaOCl was measured spectrophotometrically with G3P as substrate in the presence of NAD^+^ as coenzyme. NADH production was monitored in function of time as an absorbance increase at 340 nm^10^. The remaining GapDH activity was calculated from the slope in the kinetic curves as described previously^22^. Treatment with 200 µM H_2_O_2_ alone did not affect GapDH activity, but 500 µM H_2_O_2_ resulted in a 60% GapDH activity decrease. The enzyme was fully inactivated with 1 mM H_2_O_2_ **(**Fig. 4A **)**. GapDH inactivation with 1 mM H_2_O_2_ was 65% irreversible while 35% activity could be recovered with 10 mM DTT **(**Fig. 4C **)**. This suggests that the GapDH active site was rapidly overoxidized to Cys sulfonic acid by H_2_O_2_, but part of the enzyme was also reversible inactivated perhaps due to an intramolecular disulfide between Cys153 and Cys157 **(**Fig. 4D **)**. This intramolecular disulfide has been detected also in other GapDH homologs of *E*. *coli* and *Bacillus subtilis* under oxidative stress^32, 33^. Using Orbitrap mass spectrometry, we could confirm the formation of the Cys153-sulfonic acid and of the intramolecular disulfide between Cys153 and Cys157 after exposure to 1 mM H_2_O_2_ **(**Figure S3A **)**. In agreement with the activity assays, the overoxidized Cys153-peptide was detected at higher abundance compared to the intramolecular disulfide peptide.

Next, we analyzed whether *S*-mycothiolation can prevent overoxidation of the GapDH active site. Thus, the inhibition of GapDH activity and its reversibility was analyzed in the presence of H_2_O_2_ and MSH. GapDH was pre-treated with a 10-molar excess of MSH before it was subjected to 200 µM, 500 µM and 1 mM H_2_O_2_. Of note, GapDH inactivation by H_2_O_2_ and MSH was faster compared to H_2_O_2_ alone since 200–500 µM H_2_O_2_ resulted in a 40–75% GapDH activity decrease in the presence of MSH **(**Fig. 4B **)**. The treatment with 1 mM H_2_O_2_ and MSH lead to a complete enzyme inactivation which was comparable to the inactivation by H_2_O_2_ alone. However, GapDH inactivation by H_2_O_2_ and MSH was 80% reversible with 10 mM DTT, which indicates that Cys153 is *S*-mycothiolated in the presence of MSH and H_2_O_2_. The *S*-mycothiolated Cys153 peptide could be verified by mass spectrometry and by non-reducing MSH-specific Western blot analysis **(**Figs 4E, S3B and S4A **)**. In addition, the intramolecular Cys153-SS-Cys157 disulfide peptide was also detected by mass spectrometry **(**Figure S3B **)**. These results provide evidence for the high reactivity of the nucleophilic active site Cys153 towards H_2_O_2_, its vulnerability to overoxidation and the protection from overoxidation by *S*-mycothiolation **(**Fig. 4E **)**. Moreover, our results support that GapDH inactivation by *S*-mycothiolation occurs faster compared to overoxidation by H_2_O_2_ alone which was observed in the activity assays with 200 and 500 µM H_2_O_2_ **(**Fig. 4A,B **)**. Thus, *S*-mycothiolation can efficiently prevent the overoxidation of the GapDH active site.

**Figure 5:**
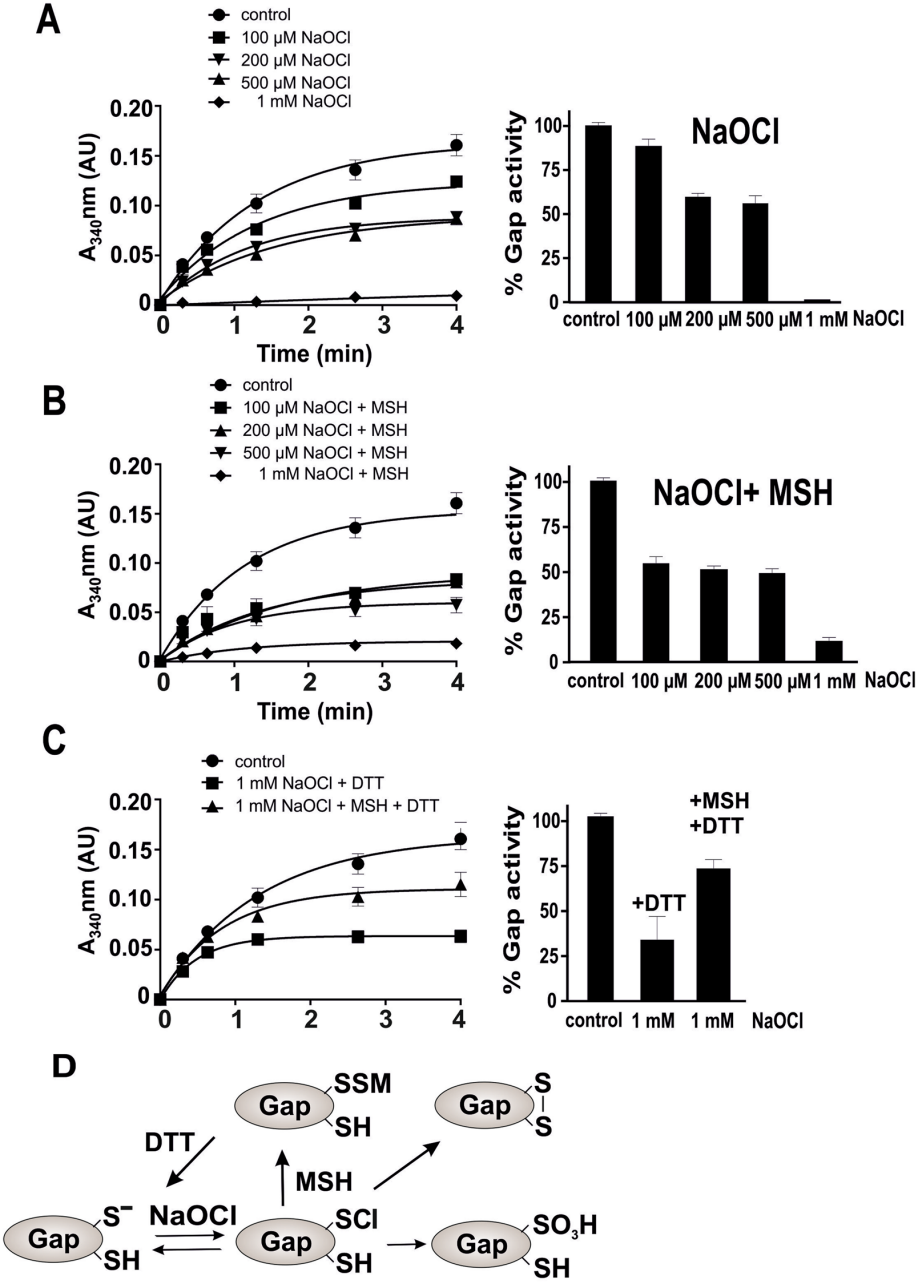
*S*-mycothiolation protects GapDH against overoxidation under NaOCl stress *in vitro*. **(A**,**B)** The NAD^+^-dependent GapDH activity was determined in a spectrophotometric assay by monitoring NADH generation during G3P oxidation at 340 nm. Inactivation of the GapDH activity was performed with 100, 200, 500 µM and 1 mM NaOCl **(A)** without or **(B)** with MSH pre-treatment. **(A**,**C)** GapDH inactivation with 1 mM NaOCl alone is mostly irreversible due to the overoxidation of the active site to Cys sulfonic acid. **(B**,**C)** GapDH activity is reversibly inhibited due to S-mycothiolation with 1 mM NaOCl and MSH and could be reactivated by 10 mM DTT. The *S*-mycothiolation of Gap was confirmed by MSH-specific Western blots **(**Figure S4). (**D)** These results suggest that the GapDH active site Cys is chlorinated by NaOCl alone to form Cys-sulfenylchloride (-SCl) that reacts further to form Cys sulfonic acid and intramolecular disulfides in the absence of MSH. GapDH is protected against overoxidation by *S*-mycothiolation of the active site Cys in the presence of MSH. All data represent mean values of three independent replicate experiments and the error bars given were calculated as standard error of the mean (SEM).

However, *S*-mycothiolation of the GapDH active site Cys153 was observed *in vivo* under conditions of NaOCl stress. Thus, we analyzed GapDH inactivation with different NaOCl concentrations in the absence or presence of MSH. The incubation of GapDH with 100 µM NaOCl did not affect its activity and concentrations of 200–500 µM led to a 40% activity decrease **(**Fig. 5A **)**. GapDH was fully inactivated with 1 mM NaOCl. Interestingly, the treatment of GapDH with 1 mM NaOCl was also partly (30%) reversible with 10 mM DTT **(**Figure 5C **)**. This suggests that GapDH inactivation must be caused by both, irreversible overoxidation of Cys153 and reversible Cys153-SS-Cys157 intramolecular disulfide bond formation under NaOCl stress **(**Fig. 5D **)**. Using Orbitrap mass spectrometry, we could confirm the overoxidation of GapDH as main modification which occurred in this case at Cys153 and Cys157. The intramolecular disulfide between Cys153 and Cys157 was also detected under NaOCl stress, but at lower abundance **(**Figure S3C **)**. In conclusion, GapDH is subject to overoxidation and intramolecular disulfide formation under both, H_2_O_2_ and NaOCl treatment *in vitro*.

To investigate whether *S*-mycothiolation can prevent the overoxidation of the active site by NaOCl, we repeated the GapDH activity assays above and pretreated the enzyme with 10-fold molar excess of MSH prior to NaOCl exposure. Exposure of GapDH to 100 µM NaOCl resulted in 35% activity decrease while 200–500 µM NaOCl caused 50% enzyme inactivation **(**Fig. 5B **)**. Treatment with 1 mM NaOCl in the presence of MSH led to 90% inactivation. Thus, it appears that GapDH inactivation with 100–500 µM NaOCl and MSH is faster compared to inactivation with NaOCl alone. GapDH inactivation by 1 mM NaOCl and MSH was almost completely reversible, since about 75% GapDH activity could be recovered with DTT. These results indicate that the GapDH active site should be protected against overoxidation by *S*-mycothiolation under NaOCl treatment in the presence of MSH. The *S*-mycothiolation of GapDH after NaOCl treatment was verified by MSH-specific Western blots and both S-mycothiolated Cys153 and Cys157 peptides were identified by mass spectrometry **(**Figures S3D and S4B **)**. Apart from *S*-mycothiolation, we identified less abundant Cys153-SS-Cys157 intramolecular disulfides under NaOCl stress in the presence of MSH. In conclusion, our activity assays provide evidence that the *S*-mycothiolation pathway occurs faster compared to the overoxidation under both, H_2_O_2_ and NaOCl treatment *in vitro*. Thus, *S*-mycothiolation can efficiently protect the active site against overoxidation and irreversible inactivation under H_2_O_2_ and NaOCl stress *in vitro* (Figs 4D and 5D). In addition, intramolecular disulfides were detected under both, H_2_O_2_ and NaOCl treatment in the presence and absence of MSH as an additional redox-regulatory mechanism of GapDH.

### Reactivation of *S*-mycothiolated GapDH requires the Mrx1/MSH/Mtr and Trx/TrxR electron transfer pathways

**Figure 6:**
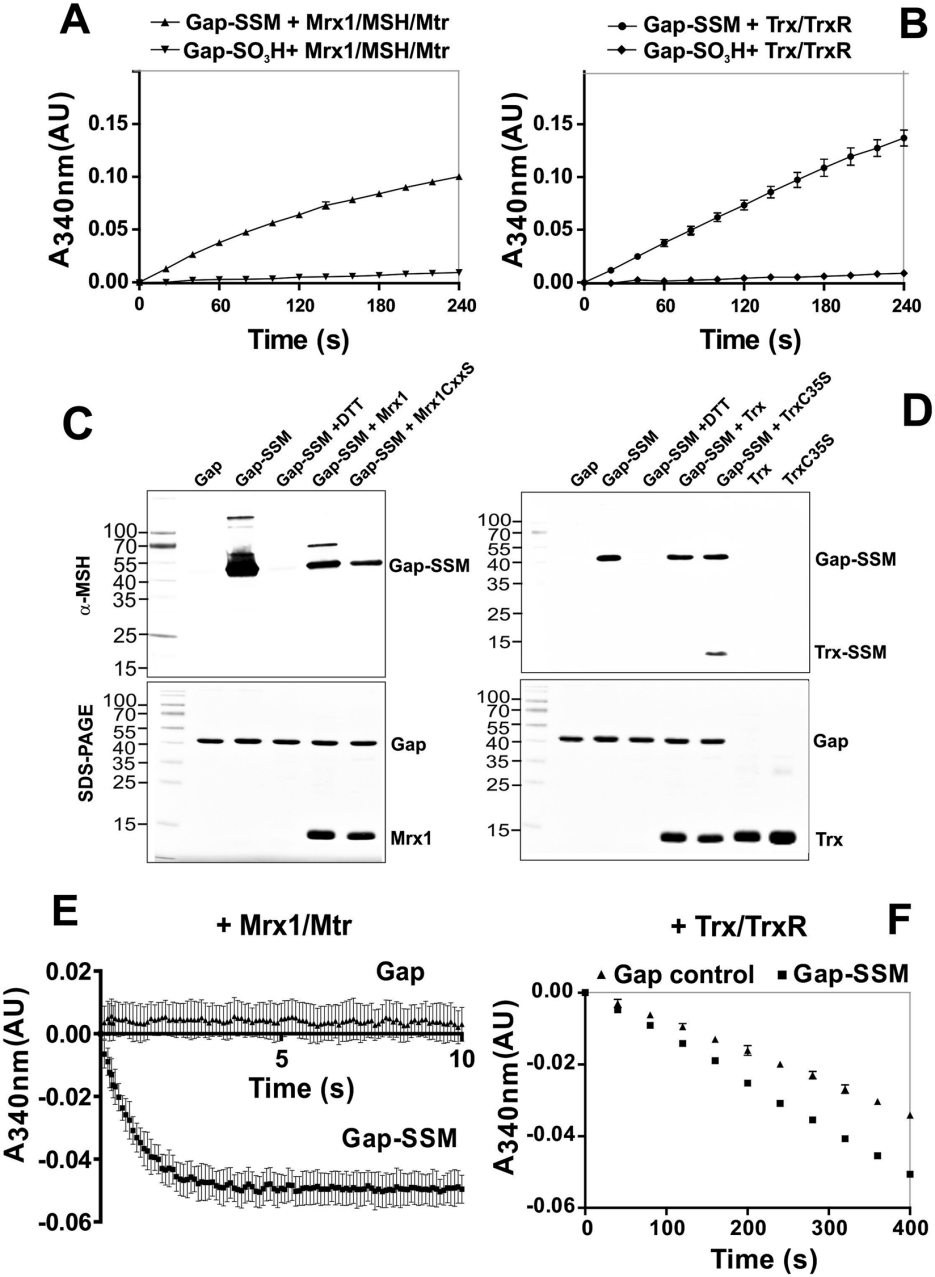
Re-activation of *S*-mycothiolated GapDH by the Trx/TrxR and Mrx/MSH/Mtr electron transfer pathways. **(A**,**B)** GapDH activity could be restored after demycothiolation of *S*-mycothiolated GapDH with Mrx1 and Trx as shown by NADH production in the G3P oxidation assay. In contrast, overoxidized GapDH that was treated with 10 mM H_2_O_2_ alone could not be reactivated by the Mrx1 and Trx pathways. **(C**,**D)** MSH-specific non-reducing Western blot analysis confirmed the *S*-mycothiolation of GapDH *in vitro* and its demycothiolation by the Mrx1 and Trx pathways. The transfer of MSH to the Trx resolving Cys mutant TrxC35S is shown. **(E**,**F)** The Mrx1/MSH/Mtr and Trx/TrxR electron transfer pathways both reduce *S*-mycothiolated GapDH with different reaction rates as revealed by progress curves of NADPH consumption. The demycothiolation of GapDH by the Mrx1-pathway was faster compared to the Trx-pathway. All data represent mean values of three independent replicate experiments and the error bars given were calculated as standard error of the mean (SEM).

Previous studies have demonstrated that both, the Mrx1 and Trx electron transfer pathways can function in reduction of the *S*-mycothiolated peroxidase Mpx *in vitro* ^13, 34^. Moreover, de-mycothiolation by Mrx1 was shown to operate faster via a monothiol reaction mechanism compared to the reduction via Trx using a dithiol mechanism. Thus, we were interested to see if the Mrx1 and/or Trx electron transfer pathways could function in the reduction of *S*-mycothiolated GapDH resulting in recovery of its glycolytic activity *in vitro*. Regeneration of GapDH activity using Mrx1 and/or Trx should work only with the *S*-mycothiolated protein, but not with the overoxidized GapDH protein. Thus, the GapDH activity assay was performed after treatment of *S*-mycothiolated and overoxidized GapDH with the Mrx1 and Trx pathways **(**Fig. 6A,B **)**. The regeneration of GapDH activity after Mrx1 and Trx reduction was followed by monitoring the NADH production at 340 nm. The results showed that both, Mrx1 and Trx can catalyze the reduction of *S*-mycothiolated GADPH to regenerate GapDH activity *in vitro* **(**Fig. 6A,B **)**. In contrast, Mrx1 and Trx could not restore the activity of overoxidized GapDH that was irreversibly inactivated using 10 mM H_2_O_2_ alone **(**Fig. 6A,B **)**. To verify the de-mycothiolation of *S*-mycothiolated GapDH by Mrx1 and Trx, we performed a MSH-specific Western blot analysis **(**Fig. 6D **)**. The results showed that Mrx1 and the Mrx1 resolving Cys mutant (Mrx1C15S) could reduce the GapDH MSH-mixed disulfide in this de-mycothiolation assay as shown by a decreased intensity of the *S*-mycothiolated GapDH band. Similarly, the reduction of GapDH-SSM by Trx and the Trx resolving Cys mutant (TrxC35S) are shown using the MSH-specific Western bot analysis. Here, the transfer of MSH to the Trx active site was clearly visible **(**Fig. 6D **)**.

Next, we analyzed whether there is a catalytically relevant reduction mechanism of GapDH by the Mrx1 and Trx electron pathways which can be monitored by NADPH consumption. The Mrx1/MSH/Mtr/NADPH and Trx/TrxR/NADPH pathways were reconstituted *in vitro* using *S*-mycothiolated GapDH as substrate and NADPH consumption was followed over time in progress curves. First, we analyzed reduction of *S*-mycothiolated GapDH with the Mrx1/MSH/Mtr pathway at 340 nm. However, we failed to see any higher NADPH consumption rate using the Mrx1 electron transfer pathway. We concluded that reduction of *S*-mycothiolated GapDH with the Mrx1 pathway might be too fast and already finished before we started the measurement. Therefore, we decided to shift to a stopped flow device with a 2 ms mixing time. Under the same conditions, we found that most NADPH was already consumed within 5 seconds **(**Fig. 6E **)**. In contrast, de-mycothiolation of *S*-mycothiolated GapDH could be measured in the Trx-coupled assay using a spectrophotometer. Here, NADPH is much slower consumed within 100 to 400 seconds. The NADPH consumption rate using Trx was higher for *S*-mycothiolated GapDH compared to the reduced GapDH control, indicating that Trx is able to reduce *S*-mycothiolated GapDH **(**Fig. 6F **)**. In conclusion, our results demonstrate that both Mrx1 and Trx can provide electrons for GapDH de-mycothiolation. However, Mrx1 reduces the MSH mixed disulfide of GapDH much faster compared to Trx. Thus, our results show that *S*-mycothiolation of GapDH can efficiently function in protection of the active site against overoxidation and can be reversed using both, the Mrx1 and Trx pathways *in vitro*.

## Discussion

Protein *S*-mycothiolation is a widespread and emerging redox modification in Actinomycetes and functions in redox regulation and thiol-protection against overoxidation to Cys sulfonic acids under conditions of NaOCl stress^9, 10^. Hypochloric acid (HOCl) is encountered by the pathogens *C*. *diphtheriae* and *M*. *tuberculosis* particularly during infections. HOCl is generated in neutrophils by the enzyme myeloperoxidase (MPO) with the aim to kill pathogenic bacteria^35, 36^. HOCl is a strong thiol-oxidant and chlorinating agent that reacts with Cys residues to sulfenylchlorides (-SCl) and further to protein disulfides^37, 38^, such as *S*-thiolations as we observed in Gram-positive bacteria^4^.

We identified 25 *S*-mycothiolated proteins in *C*. *glutamicum* ^10^ and 26 proteins in *C*. *diphtheriae* while protein *S*-mycothiolation was more abundant in *M*. *smegmatis* ^9^ with 58 identified proteins under NaOCl stress. The different numbers of *S*-mycothiolated proteins might be related to the different MSH contents between corynebacteria and mycobacteria^39^. Mycobacteria produce high levels of up-to 20 mM MSH^5^ and we recently estimated 6 µmol/g rdw MSH in *M*. *smegmatis* ^9^. However, the MSH-levels determined in *C*. *diphtheriae* are 20-fold lower with 0.3 µmol/g rdw according to this work and previous studies^5^. Due to the low MSH-content, the number of *S*-mycothiolated proteins might be lower in *C*. *diphtheriae* and *C*. *glutamicum* compared to mycobacteria. This indicates that in addition to MSH, unknown alternative LMW thiols might function in corynebacteria to maintain the thiol-redox homeostasis and to protect proteins by alternative *S*-thiolations. Recent studies further suggest that overexpression of the mycothiol disulfide reductase (Mtr) under oxidative stress conditions could play an important role in the maintenance of the redox homeostasis by increasing the levels of reduced MSH^40^. In *M*. *tuberculosis*, MSH and the alternative LMW thiol ergothioneine (EGT) have been shown to be critical for redox homeostasis, energy metabolism and virulence and mutants deficient in MSH or EGT biosynthesis showed overlapping responses in the transcriptome^41, 42^. The EGT levels were also elevated in a *M*. *smegmatis mshA* mutant^43^. Thus, it remains to be elucidated whether EGT plays also a role as alternative LMW thiol in corynebacteria. In addition, it is also possible that the lower intracellular MSH level and the lower level of protein *S*-mycothiolation in corynebacteria is related to their 2–3-fold smaller genome size compared to mycobacteria.

The comparison of the functions and conservation of all identified *S*-mycothiolated proteins across Actinomycetes indicates that these are involved in a variety of cellular pathways. *S*-mycothiolated proteins participate in energy metabolism, fatty acid and mycolic acid biosynthesis, nucleotide, cofactor, mycothiol and amino acid biosynthesis, redox regulation, detoxification, transcription and translation. Some *S-*mycothiolated proteins are conserved and essential targets for *S-*thiolation across Gram-positive bacteria, such as thiol-peroxidases and peroxiredoxins (Tpx, AhpC), ribosomal proteins (RpsM, RplC), the IMP dehydrogenase (GuaB), the myo-inositol-1-phosphate synthase (Ino1), the methionine synthase (MetE), and the conserved glycolytic GapDH.

These conserved targets for *S*-mycothiolations overlap also with conserved *S*-bacillithiolated proteins in *Firmicutes*, such as *Bacillus* and *Staphylococcus* species^11, 44^. Of note, the methionine synthase MetE is the most abundant *S*-bacillithiolated metabolic enzyme in *B*. *subtilis*, while GapDH represents the major *S*-bacillithiolated protein in *S*. *aureus* ^21, 33^. GapDH of *S*. *aureus* contributes with 4% Cys abundance to the total Cys proteome and is the most abundant Cys protein in the proteome. In *C*. *diphtheriae*, GapDH represents also the most abundant *S*-mycothiolated protein, but contributes only with 0.75% Cys abundance to the total Cys proteome. In *C*. *glutamicum*, the major targets for *S*-mycothiolation are the maltodextrin phosphorylase MalP and the thiol-peroxidase Tpx and it was shown that *S*-mycothiolation inhibited the activities of MalP and Tpx^10, 15^. Thus, it seems that abundant redox-sensitive metabolic enzymes are the main targets for inactivation by *S*-thiolations in different bacteria. The different abundances of the *S*-mycothiolated MetE, MalP and GapDH in corynebacteria most likely depend on the different minimal growth media used for bacterial cultivations.

In addition, we found that many *S*-mycothiolated proteins of *C*. *diphtheriae* contain a high number of Cys residues explaining their susceptibility to oxidative inactivation. The glycolytic GapDH was *S*-mycothiolated at its active Cys153 residue that is known to be highly susceptible to oxidation by H_2_O_2_^45–48^. GapDH is a well-known and conserved target for redox-regulation and *S*-glutathionylation in response to oxidative stress in several prokaryotic and eukaryotic organisms, including bacteria, malaria parasites, yeast, plants and human cell^19, 20, 49^. GapDH inactivation in response to oxidative stress has been shown to reprogram central carbon metabolism and to re-direct the glycolytic flux into the pentose phosphate pathway (PPP) to increase NADPH production under conditions of high demands for reducing equivalents^50, 51^. Thus, the goal of the GapDH inactivation by *S*-thiolation could be metabolic adaptation to provide more NADPH as reducing power in the cell under oxidative stress. In fact, a change of the global carbon flux was shown in *E*. *coli* under superoxide and H_2_O_2_ stress leading to an increased NADPH/NADH ratio^52, 53^. Post-translational thiol-modifications play a key role in this metabolic adaptation to oxidative stress in different organisms and can change enzyme functions to re-configurate central carbon metabolism which confers high metabolic plasticity^50, 51^.

In this study, we have asked the question whether *S*-mycothiolation can function in thiol-protection and redox-regulation of GapDH activity in *C*. *diphtheriae* under H_2_O_2_- and NaOCl stress. To address this question, GapDH was inactivated with H_2_O_2_ and NaOCl in the absence and presence of MSH to analyze the kinetics of the irreversible overoxidation and *S*-mycothiolation pathways *in vitro*. The kinetic curves of H_2_O_2_ and NaOCl-dependent GapDH inactivation showed that the majority (65%) of the glycolytic activity is rapidly irreversibly inhibited with 1 mM H_2_O_2_ and NaOCl without pre-exposure to MSH. The mass spectrometry data confirmed the overoxidation of the active site Cys153 with H_2_O_2_ and NaOCl alone. In addition, 35% of GapDH activity was reversibly inhibited by 1 mM H_2_O_2_ and NaOCl alone due to an intramolecular Cys153-SS-Cys157 disulfide that was identified using mass spectrometry. In presence of MSH, GapDH inactivation by H_2_O_2_ and NaOCl was faster due to *S*-mycothiolation which was fully reversible with DTT and confirmed also by MSH-specific Western blot analysis. This indicates that the GapDH overoxidation can be prevented by the faster *S*-mycothiolation. These results are in agreement with kinetic measurements performed for the related GapDH homolog of *S*. *aureus* ^21^. The *S*. *aureus* GapDH was highly susceptible to overoxidation in the presence of H_2_O_2_ and NaOCl which could be prevented by *S*-bacillithiolation^21^. Interestingly, the comparison of the kinetics for the dose-dependent inactivation suggests that the *S*. *aureus* GapDH enzyme is more sensitive to oxidative inactivation compared to GapDH of *C*. *diphtheriae* since lower H_2_O_2_ and NaOCl doses inhibited *S*. *aureus* GapDH activity. This higher sensitivity of *S*. *aureus* GapDH may be due to the fact that Cys157 is replaced by a serine in the otherwise highly conserved C_153_TTNC_157_ motif^21^, so there is no possibility of intramolecular disulfide formation to prevent overoxidation **(**Figure S2 **)**. The active site Cys in *Homo sapiens* GapDH was demonstrated to provide a proton relay mechanism that determines H_2_O_2_-sensitivity of GapDH for oxidation^22^. On the other hand, *S*. *aureus* GapDH contains a threonine in position 243 instead of the otherwise conserved valine, which compensates for the disappearance of the oxidation sensitivity in the C157S mutant. This was demonstrated with the *Homo sapiens* GapDH C156S mutant, where an additional V243T mutation restores the oxidation sensitivity^22^. Our results strongly suggest that the second conserved cysteine might play an important role for oxidation sensitivity of GapDH and prevents overoxidation through intramolecular disulfide formation. Further studies are required to confirm whether Cys157 or other structural features make a difference in the sensitivity of GapDH to overoxidation and *S*-mycothiolation.

The strong H_2_O_2_ reactivity of the GapDH active site thiolate was recently shown to depend on a specific H_2_O_2_ binding pocket, transition state stabilization, and a dedicated proton relay mechanism promoting hydroxyl leaving-group departure^20, 22^. This proton relay also determines the preferred modification by *S*-glutathionylation in eukaryotic organisms which requires the initial formation of a sulfenic acid at Cys153 followed by reaction with GSH to form the mixed disulfide. This proton relay explains why GapDH of *C*. *diphtheriae* is a preferred target for *S*-mycothiolation under H_2_O_2_ and NaOCl. Our results confirmed the reactivity of the GapDH active site Cys towards H_2_O_2_- and NaOCl-dependent oxidation and the preference for formation of S-thiolations as observed in other GapDH homologs.

The reduction of *S*-mycothiolated proteins was previously shown to require both, the Mrx1 and Trx pathways for the regeneration of the activities of Mpx and MsrA *in vitro* ^12, 13, 16, 34, 54^. Mpx and MsrA form intramolecular disulfides and *S-*mycothiolations under H_2_O_2_ treatment *in vitro* that are reduced by the Trx and Mrx1 pathways. Here, we have shown that reduction and re-activation of *S*-mycothiolated GapDH also requires both, the Mrx1 and the Trx pathway *in vitro*. We have further shown that Mrx1 is much faster than Trx in reduction of *S*-mycothiolated GapDH. Thus, Mrx1 can take over the role of Trx, especially when Trx, as a ubiquitous disulfide reductase, is busy with reducing other non-native disulfides upon recovery from oxidative stress. Mrx1 efficiently functions in regeneration of GapDH activity to restore cellular growth and survival. The overlapping roles of Mrx1 and Trx in demycothiolation at different reaction rates were recently shown for Mpx recycling^13^. In agreement with our GapDH results, Mpx de-mycothiolation was also about two orders of magnitude more efficient with the Mrx1 system. De-mycothiolation of Mpx by Mrx1 occurs via a monothiol mechanism, which generates MSSM, and de-mycothiolation by Trx occurs via a dithiol-mechanism, generating oxidized Trx and reduced MSH. Both results suggest Mrx1 is the primary de-mycothiolating enzyme in Actinomycetes, with Trx having only a residual contribution. Under these premises, Trx would only be able to take over the role of Mrx1 if the concentration of reduced MSH is limiting, or if Trx is present at a much higher concentration than Mrx1 inside the cell. In conclusion, de-mycothiolation using the Mrx1 and Trx pathways may be a common mechanism to recover after oxidative stress when the pentose pathway has again produced enough NADPH to ensure the regeneration of oxidized Cys residues.

Similar to our studies, the de-glutathionylating activity of Trx was shown for GapDH isoform 1 (AtGapC1) from *A*. *thaliana* that could be reactivated by glutaredoxin C and less efficiently by thioredoxin *in vitro* ^23^. De-glutathionylation using Trx1 and Grx1 was also demonstrated for other GapDH homologs and *S*-glutathionylated enzymes in the malaria parasite, *Plasmodium falciparum* and in yeast cells^49, 55, 56^. In *C*. *glutamicum*, overexpression of the MSH disulfide reductase Mtr resulted in a higher reduced level of MSH and increased activities of several redox-enzymes, including Mpx, MsrA, Trx, and Mrx1^40^. Thus, future research should be directed to explore the cross-talk of the Mrx1 and Trx systems in regenerating *S*-mycothiolated proteins and MSH itself to restore the redox balance during the recovery from oxidative stress.

## Material and Methods

### Bacterial strains and growth conditions

*C*. *diphtheriae* DSM43989 was grown under vigorous agitation in Heart Infusion broth (HIB) (Difco) at 37 °C to an optical density at 580 nm (OD_580_) of 0.75–0.8. For NaOCl stress exposure, the cells were harvested, washed and re-suspended into Belitsky Minimal Medium (BMM) and further cultivated until cells have reached an OD_500_ of ~1. *E*. *coli* strains used were DH5α and BL21(DE3)p*lysS* which were cultivated in Luria-Bertani (LB) medium at 37 °C in the presence of the appropriate antibiotics, such as ampicillin (100 μg/ml) and chloramphenicol (25 µg/ml). Sodium hypochlorite (NaOCl, 15% stock solution) was purchased from Sigma Aldrich. For stress experiments, *C*. *diphtheriae* cells were treated with 400 µM NaOCl for 30 min.

### Identification of *S*-mycothiolated peptides using LTQ-Orbitrap Velos mass spectrometry

N-ethylmaleimide (NEM)-alkylated protein extracts were prepared from *C*. *diphtheriae* cells exposed to 400 µM NaOCl for 30 min and separated by 15% non-reducing SDS-PAGE followed by tryptic in-gel digestion and LTQ-Orbitrap-Velos mass spectrometry as described^10^. Post-translational thiol-modifications of proteins were identified by searching all MS/MS spectra in “dta” format against the *C*. *diphtheriae* target-decoy protein sequence database extracted from UniprotKB release 12.7 (UniProt Consortium, Nucleic acids research 2007, 35, D193-197) using Sorcerer™-SEQUEST® (Sequest v. 2.7 rev. 11, Thermo Electron including Scaffold 4.0, Proteome Software Inc., Portland, OR). The SEQUEST search parameters and thiol-modifications were used as described^10^ using the following parameters: parent ion mass tolerance 10 ppm and fragment ion mass tolerance 1.00 Da. Two tryptic miscleavages were allowed. Methionine oxidation (+15.994915 Da), cysteine alkylation (+125.04767 Da for NEM), *S-*cysteinylations (+119.004099 Da for C3H7NO2S) and *S-*mycothiolations (+484.13627 Da for MSH) were set as variable post-translational modifications in the Sequest search. Sequest identifications required ΔCn scores of >0.10 and XCorr scores of >2.2, 3.3 and 3.75 for doubly, triply and quadruply charged peptides. Neutral loss precursor ions characteristic for the loss of myo-inositol (−180 Da) served for verification of the *S-*mycothiolated peptides. The mass spectrometry (MS) proteomics datasets (MS raw files and Scaffold files) are deposited into the ProteomeXchange database via the PRIDE partner repository with the dataset identifier PXD003321.

Mass spectrometry of the H_2_O_2_-treated overoxidized GapDH was performed after in-gel tryptic digestion using nLC-MS/MS by an Orbitrap fusion as described previously^57^.

### Monobromobimane-labelling and HPLC-thiol metabolomics analysis

Cells were cultivated in HIB medium and transferred to BMM medium for the NaOCl stress experiments as described above. Thiol-labelling using monobromobimane (mBBr) was performed as described previously^11^. The mBBr-labelled thiols were separated by reverse phase chromatography and quantified by fluorescence detection using the same HPLC system as described^58^. The following gradient method was applied: 10 min 92% buffer A (10% methanol, 0.25% acetic acid, pH 3,9) supplemented with 8% buffer B (90% methanol, 0.25% acetic acid, pH 3,9), linear increase to 40% buffer B in 10 min, constant flow of 40% buffer B for 5 min, linear increase to 90% buffer B in 5 min, washing with 100% buffer B for 2 min followed by re-equilibration with 8% buffer B for 8 min. The flow rate was constantly set to 1.5 ml min^−1^.

### Expression, cloning and purification of recombinant His_6_-tagged GapDH protein

The DIP1310 gene encoding GapDH was amplified by PCR using the primer pairs *Gap*-for (5′-GGAATTCCATATGGTGACGATTCGCGTAGGTATCA-3′) and *Gap*-rev (5′-CTAGCTAGCTTAGTGATGGTGATGGTGATGGAGACGCTCACCGACGTATTC-3′) with *C*. *diphtheriae* DSM43989 chromosomal DNA as template. The PCR product was digested with *Nhe*I and *Nde*I restriction enzymes and cloned into a similarly digested pET11b expression vector resulting in pET11b-*gapDH* that was transformed into *E*. *coli* BL21(DE3)*plysS*. The *gapDH* sequence was confirmed by DNA sequencing. For GapDH overproduction, the *E*. *coli* BL21(DE3)*plysS* strain with plasmid pET11b-*gap* was cultured in LB broth medium to an OD_600_ of 0.5 to 0.7 at 37 °C. Protein expression was induced with 1 mM IPTG (Isopropyl-β-D-1-thiogalactopyranoside) and cultivation was continued for 4 hours. Recombinant His_6_-tagged GapDH was purified by affinity chromatography using His Trap™ HP Ni-NTA columns (5 ml; GE Healthcare, Chalfont St Giles, UK) and the ÄKTA purifier liquid chromatography system (GE Healthcare) according to the instructions of the manufacturers. Purified GapDH was dialyzed against 20 mM Tris-HCl, pH 8.0 and concentrated to 20 mg/ml using Vivaspin Ultra concentrators (Sartorius, Göttingen, Germany). The cloning and purifications of recombinant His6-tagged proteins Mrx1, Mtr, Trx and TrxR were performed as described previously^59^.

### Production and purification of mycothiol

MSH was purified from *M*. *smegmatis* mc^2^155 that was grown to the late exponential phase in Middlebrook 7H9 broth with 0.05% Tween 80 and 10% oleic albumin dextrose catalase (OADC) at 37 °C as described^13^. The cells were harvested by centrifugation and disrupted using a French press (Constant Systems). The purified MSH was reduced with TCEP following several additional chromatographic steps. The concentration of MSH was determined by HPLC by correlating the MSH mBBr conjugate elution peak of an ACE 5 C18 column (Achrom) with a known standard. The sample purity was checked with Proton Nuclear Magnetic Resonance (^1^H NMR).

### Non-reducing Western blot analysis

MSH-specific Western blot analysis of the GapDH MSH-mixed disulfides were carried out using rabbit anti-MSH specific antiserum (1:1000-dilution) as described previously^13^.

### Glycolytic GapDH activity assay

GapDH was reduced before the activity assays with 10 mM DTT for 30 minutes at room temperature. Excess of DTT was removed by desalting with Micro Biospin 6 columns (Biorad). Glycolytic GapDH activity was monitored spectrophotometric at 340 nm and 25 °C by the production of NADH. The oxidation of G3P to 1,3-bisphosphoglycerate (1,3 BPG) was measured in an assay mixture containing 1.25 mM NAD^+^ and 0.25 µM GapDH in argon-flushed 20 mM Tris/HCl with 1.25 mM EDTA and 15 mM sodium arsenate as described previously^22^. After pre-incubation, the reaction was started by addition of 0.25 mM D,L-G3P. Sodium arsenate was used as a co-substrate to form unstable 1-arseno,3-phosphoglycerate. Degradation of the product allows a favorable equilibrium for measuring the rate of GapDH activity in the glycolytic forward reaction.

### Inactivation of GapDH by H_2_O_2_ and NaOCl treatment

Pre-reduced GapDH (25 µM) was incubated with different concentrations of H_2_O_2_ and NaOCl (100, 200, 500 µM, 1 mM) in the absence or presence of 1 mM MSH for 5 min at 37 °C in an assay mixture containing 1.25 mM NAD^+^ and 0.25 µM GapDH in argon-flushed 20 mM Tris-HCl with 1.25 mM EDTA and 15 mM sodium arsenate. After the removal of excess H_2_O_2_ and MSH, 0.25 mM D,L-G3P was added as substrate, GapDH activity was measured spectrophotometric by the production of NADH. The reversibility of the reaction was analyzed by measuring the GapDH activity after reduction with 10 mM DTT for 30 min.

### De-mycothiolation of GapDH by the Mrx1/MSH/Mtr and Trx/TrxR pathways

GapDH, Mrx1 and Trx were reduced before the assays with 10 mM DTT for 30 minutes at room temperature. Excess of DTT was removed by desalting with Micro Biospin 6 columns. Pre-reduced GapDH (25 µM) was pre-incubated with 10-molar excess of MSH at 37 °C for 5 min, then 100-fold molar excess of H_2_O_2_ was added and the mixture was incubated at 37 °C for 5 min. Excess of H_2_O_2_ and MSH were removed on a PD-10 desalting column (GE Healthcare). The NADPH consumption during the de-mycothiolation reactions was monitored spectrophotometrically at 340 nm and 37 °C, using argon-flushed 50 mM Hepes/NaOH, pH 8, 500 mM NaCl, 1 mM EDTA. For the reduction of *S*-mycothiolated GapDH by the Trx pathway, we used 2 µM Trx, 5 µM Trx-reductase and 250 µM NADPH in a Spectramax 340PC plate reader (Molecular Devices). For the reduction of *S*-mycothiolated GapDH by the Mrx1 pathway, we used 20 nM Mrx1, 5 µM MSH, 5 µM MSSM reductase and 250 µM NADPH in SX-20 stopped flow (Applied PhotoPhysics). After 5 min pre-incubation of this mixture at 37 °C, 60 µM mycothiolated GapDH was added to initiate the reaction. Three technical and experimental replicates were performed.

## Electronic supplementary material

Supplementary Figures

Supplemental Table S1

Supplemental Table S2

Supplemental Table S3

Supplemental Table S4
