# Supplementary material for: The glyceraldehyde-3-phosphate dehydrogenase GapDH of Corynebacterium diphtheriae is redox-controlled by protein S-mycothiolation under oxidative stress
Source: Sci Rep. 2017 Jul 10;7:5020. doi: 10.1038/s41598-017-05206-2 (PMC5504048; doi:10.1038/s41598-017-05206-2)
Supplement: Supplementary file 1 — Supplementary Figures [file 41598_2017_5206_MOESM1_ESM.pdf]

Supplementary Information for

The glyceraldehyde 3-phosphate dehydrogenase GapDH of *Corynebacterium diphtheriae* is redox-controlled by protein *S*-mycothiolation under oxidative stress

Melanie Hillion[1#], Marcel Imber[1#], Brandán Pedre[2,3,4#], Jörg Bernhardt[5#], Malek Saleh[1], Vu Van Loi[1], Sandra Maaß[5], Dörte Becher[5], Leonardo Astolfi Rosado[2,3,4,] Lorenz Adrian[6], Christoph Weise[7], Rüdiger Hell[8], Markus Wirtz[8], Joris Messens[2,3,4] and Haike Antelmann[1]*

Departments & Institutions:

[1]*Institute for Biology-Microbiology, Freie Universität Berlin, D-14195 Berlin, Germany*

[2]*Center for Structural Biology, VIB, B-1050 Brussels, Belgium*

[3]*Brussels Center for Redox Biology, B-1050 Brussels, Belgium*

[4]*Structural Biology Brussels, Vrije Universiteit Brussel, B-1050 Brussels, Belgium*

[5]*Institute for Microbiology, Ernst-Moritz-Arndt-University of Greifswald, D-17487 Greifswald, Germany*

[6]*Department Isotope Biogeochemistry, Helmholtz Centre for Environmental Research-UFZ, Leipzig, Germany*

[7]*Institute for Chemistry and Biochemistry, Freie Universität Berlin, D-14195 Berlin, Germany*

[8]*Plant Molecular Biology, Centre for Organismal Studies Heidelberg, University of Heidelberg, Heidelberg, Germany*

Running title: Redox-control of GapDH by protein *S*-mycothiolation

#These authors contributed equally to this work.

***Corresponding author:**

Haike Antelmann,Institute for Biology-Microbiology, Freie Universität Berlin,

D-14195 Berlin, Germany,Tel.+49-30-838-51221, Fax.+49-30-838-451221,

E-mail: **haike.antelmann@fu-berlin.de**

Key words: *Corynebacterium diphtheriae*/*S*-mycothiolation/GapDH/mycoredoxin

Figure S1

A

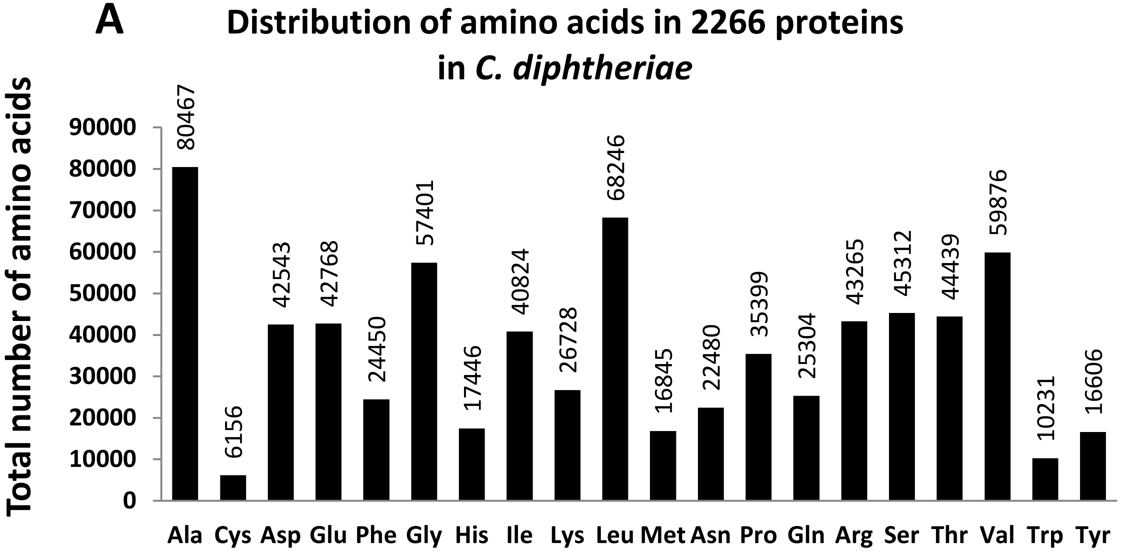
Distribution of amino acids in 2266 proteins
in *C. diphtheriae*
Total number of amino acids
90000
80000
70000
60000
50000
40000
30000
20000
10000
0
80467
6156
42543
42768
24450
57401
17446
40824
26728
68246
16845
22480
35399
25304
43265
45312
44439
59876
10231
16606
Ala
Cys
Asp
Glu
Phe
Gly
His
Ile
Lys
Leu
Met
Asn
Pro
Gln
Arg
Ser
Thr
Val
Trp
Tyr

B

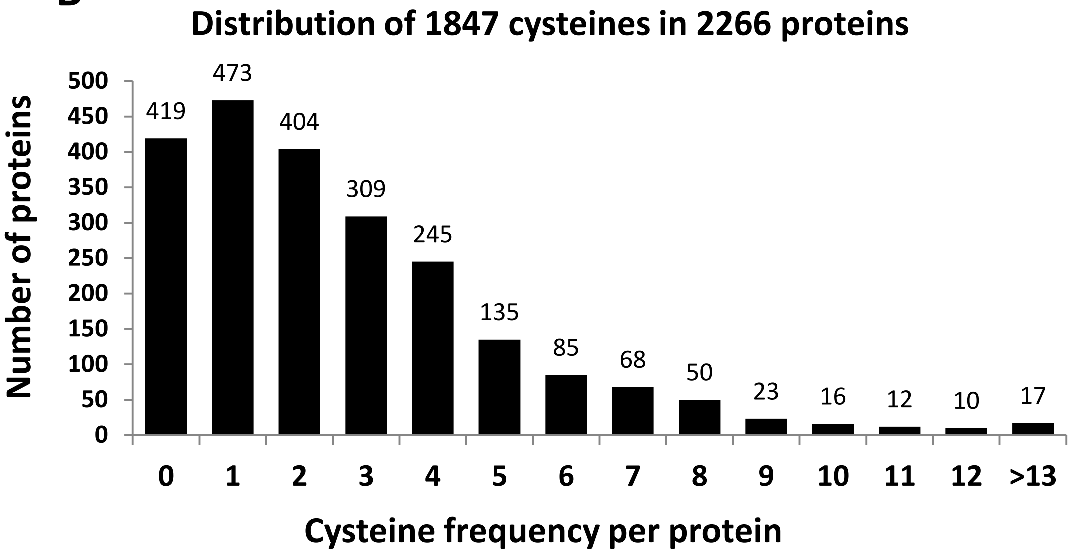
Distribution of 1847 cysteines in 2266 proteins
Number of proteins
500
450
400
350
300
250
200
150
100
50
0
419
473
404
309
245
135
85
68
50
23
16
12
10
17
0
1
2
3
4
5
6
7
8
9
10
11
12
>13
Cysteine frequency per protein

Figure S1

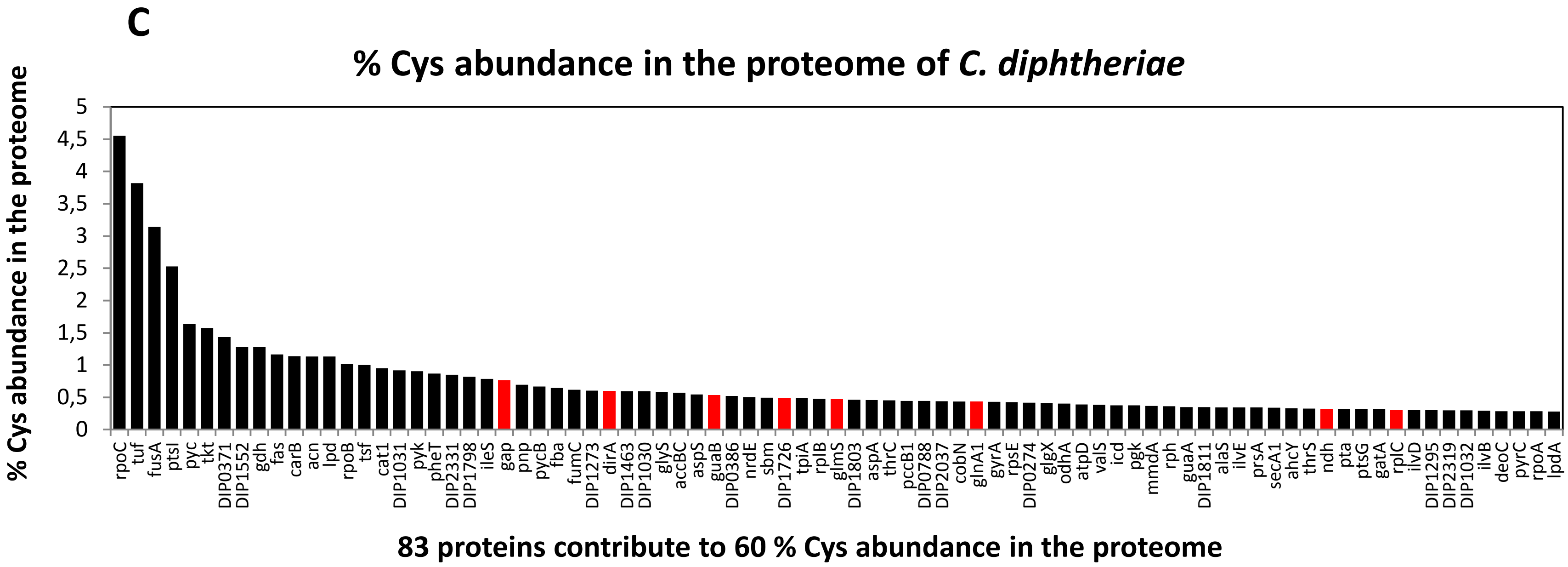

Figure S1. Distribution of amino acids (A) and Cys numbers (B) in all 2266 proteins encoded in the genome of *C. diphtheriae* and percentages of Cys abundances (C) of all proteins identified in the proteome under NaOCl stress. The 83 most abundant proteins that contribute to 60% of Cys abundances in the proteome of *C. diphtheriae* are shown indicating that the S-mycothiolated proteins Gap, DirA (AhpC), Ndh and GuaB contribute with 0.4-0.8 % to the total Cys proteome. The Cys abundances were calculated by multiplication of the spectral counts of the identified proteins with the number of Cys residues according to Table S3.

Figure S2

A

```
C.diphtheriae/1-334     1 MTIRVGINGFGRIGRNFYRAITER-----GADIEVVAINDLTDNHTLSHLLKYDSILGRLGKEVSYD-DESITVDGHRMVVTAERD-PK82
C.glutamicum/1-334      1 MTIRVGINGFGRIGRNFFRAVLER-----SDDLEVVAVNDLTDNKTLSTLLKFDSIMGRLGQEVEYD-DDSITVGGKRIAVYAERD-PK82
M.tuberculosis/1-339    1 MTVRVGINGFGRIGRNFYRALLAQQEQG-TADVEVVAANDITDNSTLAHLLKFDSILGRLPCDVGLEGDDTIVVGRAKIKALAVREGPA88

C.diphtheriae/1-334    83 NLKWGELNVDIVVESTGFFTDANAAKAHIEAGAKKVIISAPAKNEDATFVVGVNHTDYDPAKHNIISNASCTTNCLAPMAKVLDEKFGI 171
C.glutamicum/1-334     83 NLDWAAHNVDIVIESTGFFTDANAAKAHIEAGAKKVIISAPASNEDATFVYGVNHESYDPENHNVISGASCTTNCLAPMAKVLNDKFGI 171
M.tuberculosis/1-339   89 ALPWGDLGVDVVVESTGLFTNAAKAKGHLDAGAKKVIISAPATDEDITIVLGVNDDKYD-GSQNIISNASCTTNCLAPLAKVLDDEFGI 176
                                                                                               *

C.diphtheriae/1-334   172 VKGLMTTIHAYTGDQRLHDAPHR--DLRRARAAAQNIVPTSTGAAKAVALVLPELKGKLDGFAMRVPVITGSATDLTFETTKEVSAEEI 258
C.glutamicum/1-334    172 ENGLMTTVHAYTGDQRLHDAPHR--DLRRARAAAVNIVPTSTGAAKAVALVLPELKGKLDGYALRVPVITGSATDLTFNTKSEVTVESI 258
M.tuberculosis/1-339  177 VKGLMTTIHAYTQDQNLQDGPHK  DLRRARAAALNIVPTSTGAAKAIGLVMPQLKGKLDGYALRVPIPTGSVTDLTVDLSTRASVDEI 263

C.diphtheriae/1-334   259 NAAMKEAAEGELKGVLAYTEDPIVSTDIVTDAHASIFDAGLTKVIG----NQVKVVSWYDNEWGYSNQLVSLTEYVGERL-        334
C.glutamicum/1-334    259 NAAIKEAAVGEFGETLAYSEEPLVSTDIVHDSHGSIFDAGLTKVSG----NTVKVVSWYDNEWGYTCQLLRLTELVASKL-        334
M.tuberculosis/1-339  264 NAAFKAAAEGRLKGILKYYDAPIVSSDIVTDPHSSIFDSGLTKVID----DQAKVVSWYDNEWGYSNRLVDLVTLVGKSL-        339
```

B

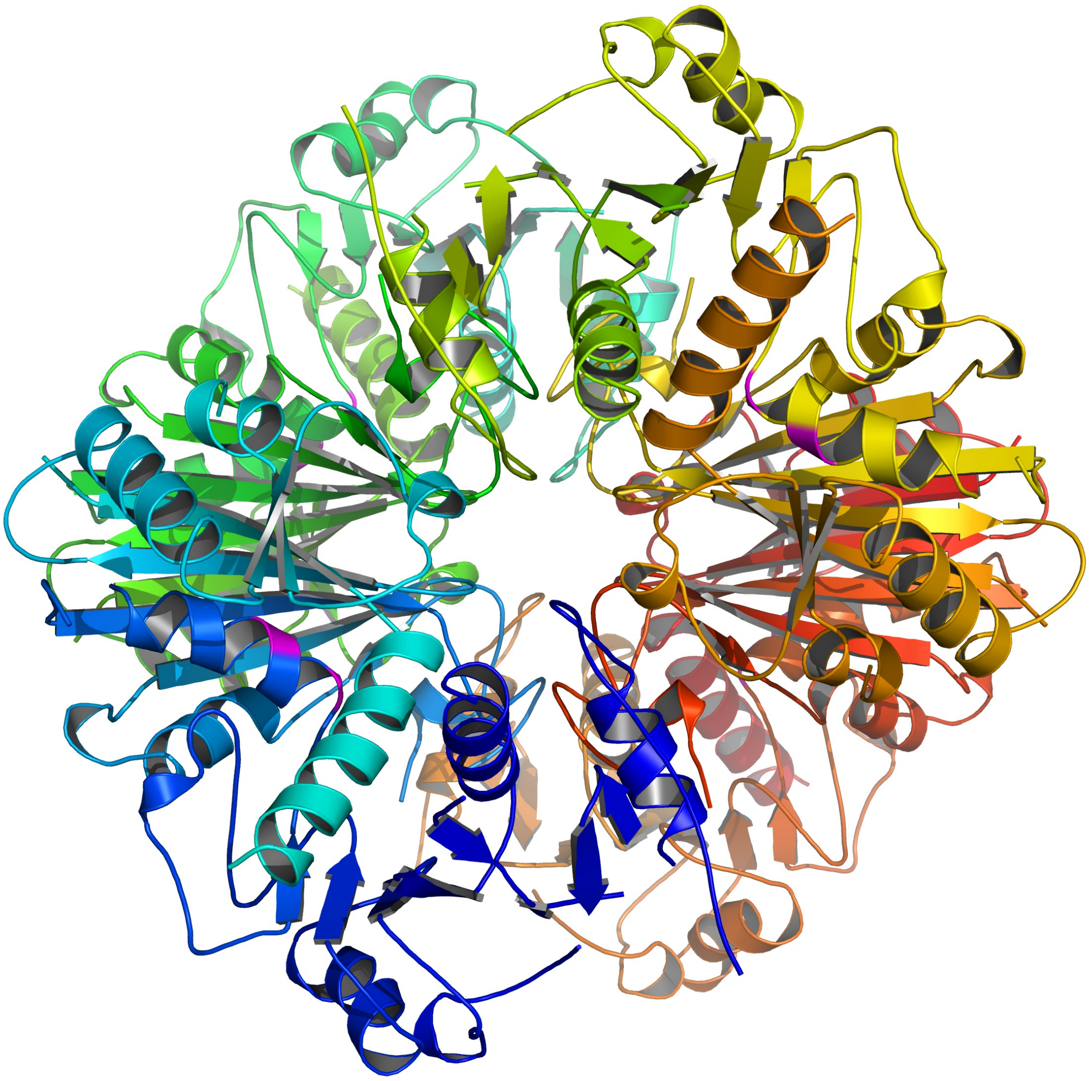

Figure S2. Multiple Gap protein sequence alignments and the Gap structure. (A) The Gap homologs from *C. diphtheriae, C. glutamicum* and *M. tuberculosis* with the Uniprot accession numbers Q6NH35, Q6NH35, P9WN83, respectively, were aligned using ClustalΩ and presented using Jalview. The intensity of the blue color gradient is based on 50% identity. The Cys residues are labelled in red and conserved amino acid residues are marked with asterisks **(B)** The structure of *C. diphtheriae* Gap tetramer was generated with Phyre2 and PyMol based on the high resolution structure of GapDH of *Thermus aquaticus* (PDB: 2G82) as template. The cysteine residues in the active site are high-lighted in magenta.

Figure S3A: Overoxidation and intramolecular disulfide formation of GapDH of *C. diphtheriae* after treatment with 1 mM H2O2

Sequence	Prob	SEQUEST XCorr	SEQUEST ΔCn	NTT	Modifications	Observed	Actual Mass	Charge	ΔDa	ΔPPM	Start	Stop	Spectrum ID
(K)HNIISNASC$_{153}$TTNC$_{157}$LAPMAK(V)	98%	6.1762	0.7077	2	Sulfonic acid (+48), Carbamidomethyl (+57)	1.047,48	2.092,94	2	0,005713	2,728	145	163	2min_H2O2.03902.03902.2.dta
(K)HNIISNASC$_{153}$TTNC$_{157}$LAPMAK(V)	98%	4.8209	0.7627	2	Sulfonic acid (+48), Carbamidomethyl (+57)	1.047,97	2.093,93	2	0,9911	-5,568	145	163	2min_H2O2.04392.04392.2.dta
(K)HNIISNASC$_{153}$TTNC$_{157}$LAPMAK(V)	98%	4.5348	0.732	2	Sulfonic acid (+48), Carbamidomethyl (+57), Met-ox (+16)	1.055,47	2.108,93	2	0,003962	1,878	145	163	2min_H2O2.03357.03357.2.dta
(K)HNIISNASC$_{153}$TTNC$_{157}$LAPMAK(V)	98%	4.4411	0.7614	2	Sulfonic acid (+48), Carbamidomethyl (+57), Met-ox (+16)	1.055,96	2.109,91	2	0,9837	-9,015	145	163	2min_H2O2.03954.03954.2.dta
(K)HNIISNASC$_{153}$TTNC$_{157}$LAPMAK(V)	98%	4.1484	0.7097	2	Sulfonic acid (+48), Carbamidomethyl (+57)	1.047,47	2.092,93	2	-0,001123	-0,5365	145	163	2min_H2O2.04181.04181.2.dta
(K)HNIISNASC$_{153}$TTNC$_{157}$LAPMAK(V)	98%	3.4969	0.7474	2	Carbamidomethyl (+57), Sulfonic acid (+48), Met-ox (+16)	1.055,47	2.108,93	2	0,00323	1,531	145	163	2min_H2O2.03587.03587.2.dta
(K)HNIISNASC$_{153}$TTNC$_{157}$LAPMAK(V)	98%	4.1668	0.67	2	Sulfonic acid (+48), Carbamidomethyl (+57)	1.046,97	2.091,92	2	-1,012	-4,599	145	163	2min_H2O2.04191.04191.2.dta
(K)HNIISNASC$_{153}$TTNC$_{157}$LAPMAK(V)	98%	3.2711	0.6594	2	Sulfonic acid (+48), Carbamidomethyl (+57), Met-ox (+16)	1.054,97	2.107,93	2	-0,9953	3,52	145	163	2min_H2O2.04885.04885.2.dta

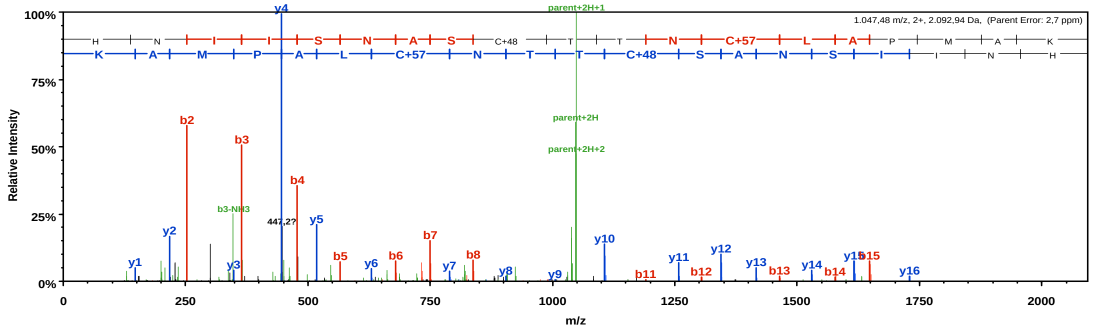

B	B Ions	B+2H	B-NH3	B-H2O	AA	Y Ions	Y+2H	Y-NH3	Y-H2O	Y
1	138,1	69,5			H	2.093,9	1.047,5	2.076,9	2.075,9	19
2	252,1	126,6	235,1		N	1.956,9	978,9	1.939,9	1.938,9	18
3	365,2	183,1	348,2		I	1.842,8	921,9	1.825,8	1.824,8	17
4	478,3	239,6	461,3		I	1.729,8	865,4	1.712,7	1.711,7	16
5	565,3	283,2	548,3	547,3	S	1.616,7	808,8	1.599,6	1.598,7	15
6	679,4	340,2	662,3	661,3	N	1.529,6	765,3	1.512,6	1.511,6	14
7	750,4	375,7	733,4	732,4	A	1.415,6	708,3	1.398,6	1.397,6	13
8	837,4	419,2	820,4	819,4	S	1.344,6	672,8	1.327,5	1.326,5	12
9	988,4	494,7	971,4	970,4	C+48	1.257,5	629,3	1.240,5	1.239,5	11
10	1.089,5	545,2	1.072,4	1.071,5	T	1.106,5	553,8	1.089,5	1.088,5	10
11	1.190,5	595,8	1.173,5	1.172,5	T	1.005,5	503,2	988,5	987,5	9
12	1.304,6	652,8	1.287,5	1.286,5	N	904,4	452,7	887,4		8
13	1.464,6	732,8	1.447,6	1.446,6	C+57	790,4	395,7	773,4		7
14	1.577,7	789,3	1.560,6	1.559,7	L	630,4	315,7	613,3		6
15	1.648,7	824,9	1.631,7	1.630,7	A	517,3		500,3		5
16	1.745,8	873,4	1.728,7	1.727,7	P	446,2		429,2		4
17	1.876,8	938,9	1.859,8	1.858,8	M	349,2		332,2		3
18	1.947,8	974,4	1.930,8	1.929,8	A	218,1		201,1		2
19	2.093,9	1.047,5	2.076,9	2.075,9	K	147,1		130,1		1

Sequence	Prob	SEQUEST XCorr	SEQUEST ΔCn	NTT	Modifications	Observed	Actual Mass	Charge	ΔDa	ΔPPM	Start	Stop	Spectrum ID
(K)HNIISNASC$_{153}$TTNC$_{157}$LAPMAK(V)	98%	5.5307	0.6896	2	Cys-SS-Cys (-2)	993,9651	1.985,92	2	-0,05062	-25,48	145	163	2min_H2O2.03842.03842.2.dta
(K)HNIISNASC$_{153}$TTNC$_{157}$LAPMAK(V)	98%	3.2124	0.745	2	Cys-SS-Cys (-2), Met-ox (+16)	1.001,96	2.001,91	2	-0,05274	-26,33	145	163	2min_H2O2.03304.03304.2.dta

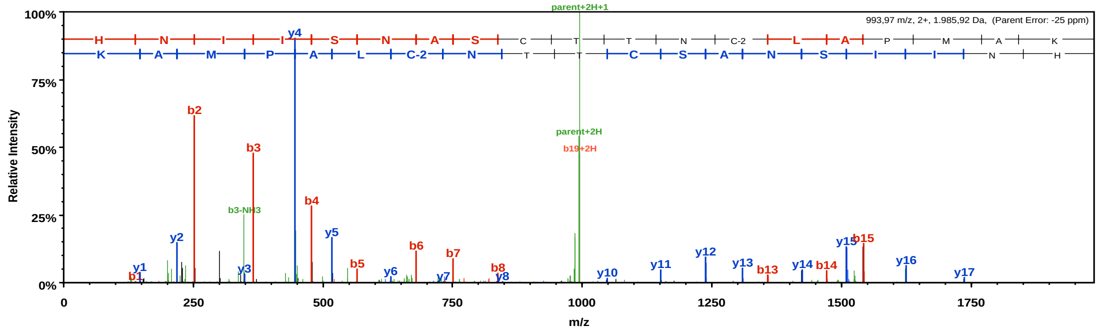

B	B Ions	B+2H	B-NH3	B-H2O	AA	Y Ions	Y+2H	Y-NH3	Y-H2O	Y
1	138,1	69,5			H	1.987,0	994,0	1.969,9	1.969,0	19
2	252,1	126,6	235,1		N	1.849,9	925,5	1.832,9	1.831,9	18
3	365,2	183,1	348,2		I	1.735,9	868,4	1.718,8	1.717,9	17
4	478,3	239,6	461,3		I	1.622,8	811,9	1.605,8	1.604,8	16
5	565,3	283,2	548,3	547,3	S	1.509,7	755,4	1.492,7	1.491,7	15
6	679,4	340,2	662,3	661,3	N	1.422,7	711,8	1.405,6	1.404,7	14
7	750,4	375,7	733,4	732,4	A	1.308,6	654,8	1.291,6	1.290,6	13
8	837,4	419,2	820,4	819,4	S	1.237,6	619,3	1.220,6	1.219,6	12
9	940,4	470,7	923,4	922,4	C	1.150,6	575,8	1.133,5	1.132,5	11
10	1.041,5	521,2	1.024,5	1.023,5	T	1.047,6	524,3	1.030,5	1.029,5	10
11	1.142,5	571,8	1.125,5	1.124,5	T	946,5	473,8	929,5	928,5	9
12	1.256,6	628,8	1.239,5	1.238,6	N	845,5	423,2	828,4		8
13	1.357,6	679,3	1.340,6	1.339,6	C-2	731,4	366,2	714,4		7
14	1.470,7	735,9	1.453,7	1.452,7	L	630,4	315,7	613,3		6
15	1.541,7	771,4	1.524,7	1.523,7	A	517,3		500,3		5
16	1.638,8	819,9	1.621,8	1.620,8	P	446,2		429,2		4
17	1.769,8	885,4	1.752,8	1.751,8	M	349,2		332,2		3
18	1.840,9	920,9	1.823,8	1.822,9	A	218,1		201,1		2
19	1.987,0	994,0	1.969,9	1.969,0	K	147,1		130,1		1

Figure S3A. LC-MS/MS fragment ion spectra for the detection of overoxidized Cys153-SO$_3$H peptide and the Cys153-SS-Cys157 intramolecular disulfide during GapDH inactivation by H_2O_2 in the absence of MSH. Gap was treated with 1 mM H_2O_2 for 2 min, followed by alkylation with IAM and tryptic digestion. The tryptic GapDH peptides were analyzed by Orbitrap mass spectrometry. The spectral counts, fragment ion spectra and tables show the formation of the several Cys153-SO$_3$H peptides as main thiol-oxidation form and less abundant intramolecular disulfides after treatment with H_2O_2 in vitro.

Figure S3B: Mycothiolation and intramolecular disulfide formation of GapDH of *C. diphtheriae* after treatment with 1 mM H2O2

Sequence	Prob	SEQUEST XCorr	SEQUEST ΔCn	NTT	Modifications	Observed	Actual Mass	Charge	ΔDa	ΔPPM	Start	Stop	Spectrum ID
(K)HNIISNASC$_{153}$TTNC$_{157}$LAPMAK(V)	84%	3.6440	0.6694	2	^ (+484), ^ (+484)	986,408	2.956,20	3	0,001839	0,6219	145	163	2min_MSH_H2O2.03715.03715.3.dta
(K)HNIISNASC$_{153}$TTNC$_{157}$LAPMAK(V)	84%	4.1553	0.6398	2	Carbamidomethyl (+57), ^ (+484)	844,0328	2.529,08	3	-0,009097	-3,596	145	163	2min_MSH_H2O2.03728.03728.3.dta
(K)HNIISNASC$_{153}$TTNC$_{157}$LAPMAK(V)	84%	3.9008	0.6129	2	^ (+484), Sulfonic acid (+48)	841,0238	2.520,05	3	0,000706	0,28	145	163	2min_MSH_H2O2.03858.03858.3.dta
(K)HNIISNASC$_{153}$TTNC$_{157}$LAPMAK(V)	84%	3.9047	0.5624	2	^ (+484), Carbamidomethyl (+57), Met-ox (+16)	849,3683	2.545,08	3	0,002518	0,9889	145	163	2min_MSH_H2O2.03291.03291.3.dta

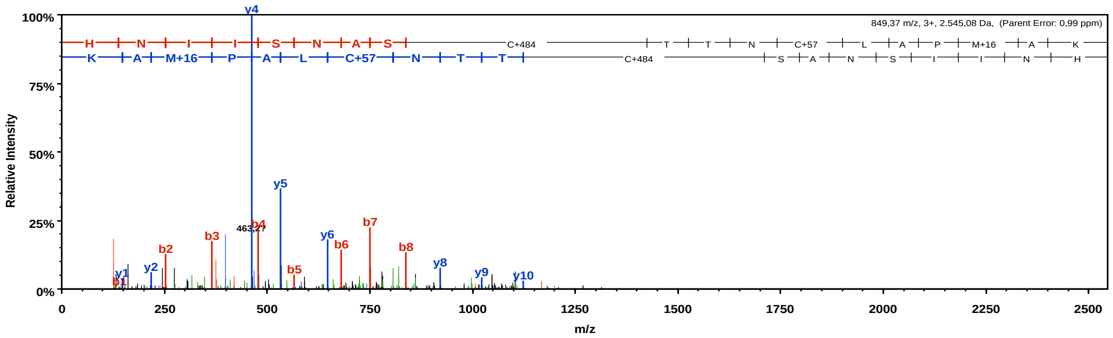

B	B Ions	B+2H	B-NH3	B-H2O	AA	Y Ions	Y+2H	Y-NH3	Y-H2O	Y
1	138,1	69,5			H	2.546,1	1.273,5	2.529,1	2.528,1	19
2	252,1	126,6	235,1		N	2.409,0	1.205,0	2.392,0	2.391,0	18
3	365,2	183,1	348,2		I	2.295,0	1.148,0	2.278,0	2.277,0	17
4	478,3	239,6	461,3		I	2.181,9	1.091,5	2.164,9	2.163,9	16
5	565,3	283,2	548,3	547,3	S	2.068,8	1.034,9	2.051,8	2.050,8	15
6	679,4	340,2	662,3	661,3	N	1.981,8	991,4	1.964,8	1.963,8	14
7	750,4	375,7	733,4	732,4	A	1.867,7	934,4	1.850,7	1.849,7	13
8	837,4	419,2	820,4	819,4	S	1.796,7	898,9	1.779,7	1.778,7	12
9	1.424,6	712,8	1.407,5	1.406,6	C+484	1.709,7	855,3	1.692,6	1.691,7	11
10	1.525,6	763,3	1.508,6	1.507,6	T	1.122,5	561,8	1.105,5	1.104,5	10
11	1.626,7	813,8	1.609,6	1.608,7	T	1.021,5	511,2	1.004,5	1.003,5	9
12	1.740,7	870,9	1.723,7	1.722,7	N	920,4	460,7	903,4		8
13	1.900,7	950,9	1.883,7	1.882,7	C+57	806,4	403,7	789,4		7
14	2.013,8	1.007,4	1.996,8	1.995,8	L	646,4	323,7	629,3		6
15	2.084,9	1.042,9	2.067,8	2.066,8	A	533,3	267,1	516,2		5
16	2.181,9	1.091,5	2.164,9	2.163,9	P	462,2	231,6	445,2		4
17	2.328,9	1.165,0	2.311,9	2.310,9	M+16	365,2	183,1	348,2		3
18	2.400,0	1.200,5	2.383,0	2.382,0	A	218,1	109,6	201,1		2
19	2.546,1	1.273,5	2.529,1	2.528,1	K	147,1	74,1	130,1		1

Sequence	Prob	SEQUEST XCorr	SEQUEST ΔCn	NTT	Modifications	Observed	Actual Mass	Charge	ΔDa	ΔPPM	Start	Stop	Spectrum ID
(K)HNIISNASC$_{153}$TTNC$_{157}$LAPMAK(V)	98%	5.7076	0.7573	2	Cys-SS-Cys (-2), Met-ox (+16)	1.001,96	2.001,91	2	-0,05359	-26,76	145	163	2min_MSH_H2O2.03428.03428.2.dta
(K)HNIISNASC$_{153}$TTNC$_{157}$LAPMAK(V)	98%	2.8711	0.6822	2	Cys-SS-Cys (-2), Met-ox (+16)	1.002,46	2.002,91	2	0,9449	-28,84 (+1 neu	145	163	2min_MSH_H2O2.03627.03627.2.dta
(K)HNIISNASC$_{153}$TTNC$_{157}$LAPMAK(V)	98%	5.3409	0.7243	2	Cys-SS-Cys (-2)	993,9639	1.985,91	2	-0,05306	-26,7	145	163	2min_MSH_H2O2.03929.03929.2.dta
(K)HNIISNASC$_{153}$TTNC$_{157}$LAPMAK(V)	98%	4.9295	0.6239	2	Cys-SS-Cys (-2)	994,4563	1.986,90	2	0,9317	-35,74 (+1 neu	145	163	2min_MSH_H2O2.04108.04108.2.dta
(K)HNIISNASC$_{153}$TTNC$_{157}$LAPMAK(V)	98%	3.4425	0.5612	2	Cys-SS-Cys (-2)	994,4588	1.986,90	2	0,9367	-33,22 (+1 neu	145	163	2min_MSH_H2O2.04397.04397.2.dta

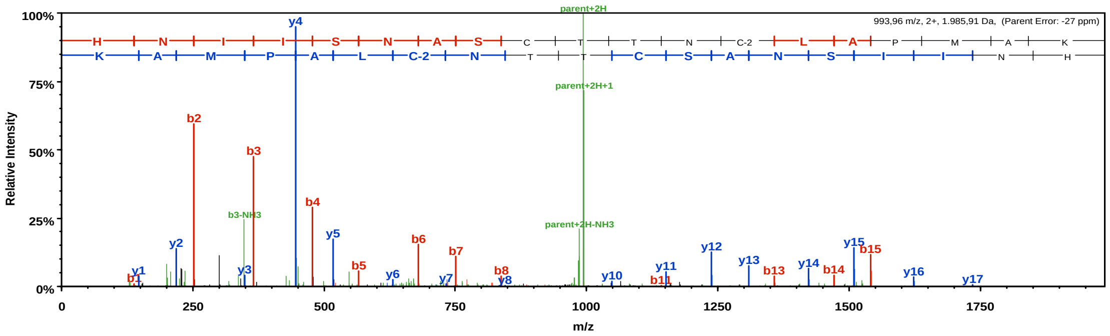

B	B Ions	B+2H	B-NH3	B-H2O	AA	Y Ions	Y+2H	Y-NH3	Y-H2O	Y
1	138,1	69,5			H	1.987,0	994,0	1.969,9	1.969,0	19
2	252,1	126,6	235,1		N	1.849,9	925,5	1.832,9	1.831,9	18
3	365,2	183,1	348,2		I	1.735,9	868,4	1.718,8	1.717,9	17
4	478,3	239,6	461,3		I	1.622,8	811,9	1.605,8	1.604,8	16
5	565,3	283,2	548,3	547,3	S	1.509,7	755,4	1.492,7	1.491,7	15
6	679,4	340,2	662,3	661,3	N	1.422,7	711,8	1.405,6	1.404,7	14
7	750,4	375,7	733,4	732,4	A	1.308,6	654,8	1.291,6	1.290,6	13
8	837,4	419,2	820,4	819,4	S	1.237,6	619,3	1.220,6	1.219,6	12
9	940,4	470,7	923,4	922,4	C	1.150,6	575,8	1.133,5	1.132,5	11
10	1.041,5	521,2	1.024,5	1.023,5	T	1.047,6	524,3	1.030,5	1.029,5	10
11	1.142,5	571,8	1.125,5	1.124,5	T	946,5	473,8	929,5	928,5	9
12	1.256,6	628,8	1.239,5	1.238,6	N	845,5	423,2	828,4		8
13	1.357,6	679,3	1.340,6	1.339,6	C-2	731,4	366,2	714,4		7
14	1.470,7	735,9	1.453,7	1.452,7	L	630,4	315,7	613,3		6
15	1.541,7	771,4	1.524,7	1.523,7	A	517,3		500,3		5
16	1.638,8	819,9	1.621,8	1.620,8	P	446,2		429,2		4
17	1.769,8	885,4	1.752,8	1.751,8	M	349,2		332,2		3
18	1.840,9	920,9	1.823,8	1.822,9	A	218,1		201,1		2
19	1.987,0	994,0	1.969,9	1.969,0	K	147,1		130,1		1

Figure S3B. LC-MS/MS fragment ion spectra for the detection of S-mycothiolated Cys153-SSM peptide and the Cys153-SS-Cys157 intramolecular disulfide during GapDH inactivation by H_2O_2 in the presence of MSH. Gap was pretreated with MSH and exposed to 1 mM H_2O_2 for 2 min, followed by alkylation with IAM and tryptic digestion. The tryptic GapDH peptides were analyzed by Orbitrap mass spectrometry. The spectral counts, fragment ion spectra and tables show the formation of the similar abundant Cys153-SSM peptides and intramolecular disulfides after treatment with H_2O_2 in vitro.

Figure S3C: Overoxidation and intramolecular disulfide formation of GapDH of *C. diphtheriae* after treatment with 1 mM NaOCl

Sequence	Prob	SEQUEST XCorr	SEQUEST ΔCn	NTT	Modifications	Observed	Actual Mass	Charge	ΔDa	ΔPPM	Start	Stop	Spectrum ID
(K)HNIISNASC$_{153}$TTNC$_{157}$LAPMAK(V)	98%	4.4466	0.7894	2	Carbamidomethyl (+57), Sulfonic acid (+48)	1.047,48	2.092,94	2	0,009131	4,361	145	163	2min_NaoCl.04169.04169.2.dta
(K)HNIISNASC$_{153}$TTNC$_{157}$LAPMAK(V)	98%	4.9546	0.7275	2	Carbamidomethyl (+57), Sulfonic acid (+48)	1.047,48	2.092,94	2	0,004493	2,146	145	163	2min_NaoCl.03953.03953.2.dta
(K)HNIISNASC$_{153}$TTNC$_{157}$LAPMAK(V)	98%	3.7396	0.7924	2	Sulfonic acid (+48), Sulfonic acid (+48), Met-ox (+16)	1.050,96	2.099,90	2	0,003573	1,701	145	163	2min_NaoCl.04281.04281.2.dta
(K)HNIISNASC$_{153}$TTNC$_{157}$LAPMAK(V)	98%	3.7680	0.7869	2	Carbamidomethyl (+57), Sulfonic acid (+48), Met-ox (+16)	1.055,47	2.108,93	2	0,001765	0,8364	145	163	2min_NaoCl.03634.03634.2.dta
(K)HNIISNASC$_{153}$TTNC$_{157}$LAPMAK(V)	98%	4.0676	0.6817	2	Carbamidomethyl (+57), Sulfonic acid (+48), Met-ox (+16)	1.055,46	2.108,91	2	-0,01801	-8,536	145	163	2min_NaoCl.04374.04374.2.dta
(K)HNIISNASC$_{153}$TTNC$_{157}$LAPMAK(V)	98%	3.7821	0.6653	2	Carbamidomethyl (+57), Sulfonic acid (+48), Met-ox (+16)	1.055,97	2.109,93	2	1,002	-0,5722	145	163	2min_NaoCl.03527.03527.2.dta
(K)HNIISNASC$_{153}$TTNC$_{157}$LAPMAK(V)	98%	2.8930	0.6701	2	Carbamidomethyl (+57), Sulfonic acid (+48)	1.047,47	2.092,93	2	-0,0003903	-0,1864	145	163	2min_NaoCl.03485.03485.2.dta
(K)HNIISNASC$_{153}$TTNC$_{157}$LAPMAK(V)	98%	2.4027	0.7319	2	Carbamidomethyl (+57), Sulfonic acid (+48), Met-ox (+16)	1.055,47	2.108,93	2	-0,002629	-1,246	145	163	2min_NaoCl.03406.03406.2.dta
(K)HNIISNASC$_{153}$TTNC$_{157}$LAPMAK(V)	98%	3.2380	0.5781	2	Carbamidomethyl (+57), Sulfonic acid (+48), Met-ox (+16)	1.055,97	2.109,92	2	0,9883	-6,818	145	163	2min_NaoCl.03830.03830.2.dta
(K)HNIISNASC$_{153}$TTNC$_{157}$LAPMAK(V)	98%	3.3962	0.5203	2	Sulfonic acid (+48), Sulfonic acid (+48)	1.042,96	2.083,90	2	0,005812	2,788	145	163	2min_NaoCl.04185.04185.2.dta
(K)HNIISNASC$_{153}$TTNC$_{157}$LAPMAK(V)	98%	2.2573	0.654	2	Sulfonic acid (+48), Sulfonic acid (+48), Met-ox (+16)	1.050,94	2.099,87	2	-0,01938	-9,223	145	163	2min_NaoCl.04808.04808.2.dta
(K)HNIISNASC$_{153}$TTNC$_{157}$LAPMAK(V)	98%	2.4716	0.5931	2	Sulfonic acid (+48), Sulfonic acid (+48), Met-ox (+16)	1.050,95	2.099,88	2	-0,01474	-7,015	145	163	2min_NaoCl.04574.04574.2.dta
(K)HNIISNASC$_{153}$TTNC$_{157}$LAPMAK(V)	98%	2.3565	0.6425	2	Sulfonic acid (+48), Sulfonic acid (+48), Met-ox (+16)	1.050,46	2.098,91	2	-0,985	8,465	145	163	2min_NaoCl.03799.03799.2.dta

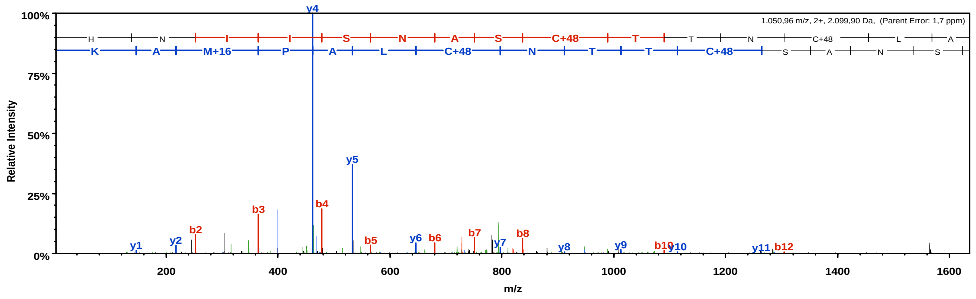

B	B Ions	B+2H	B-NH3	B-H2O	AA	Y Ions	Y+2H	Y-NH3	Y-H2O	Y
1	138,1	69,5			H	2.100,9	1.051,0	2.083,9	2.082,9	19
2	252,1	126,6	235,1		N	1.963,8	982,4	1.946,8	1.945,8	18
3	365,2	183,1	348,2		I	1.849,8	925,4	1.832,8	1.831,8	17
4	478,3	239,6	461,3		I	1.736,7	868,9	1.719,7	1.718,7	16
5	565,3	283,2	548,3	547,3	S	1.623,6	812,3	1.606,6	1.605,6	15
6	679,4	340,2	662,3	661,3	N	1.536,6	768,8	1.519,6	1.518,6	14
7	750,4	375,7	733,4	732,4	A	1.422,6	711,8	1.405,5	1.404,5	13
8	837,4	419,2	820,4	819,4	S	1.351,5	676,3	1.334,5	1.333,5	12
9	988,4	494,7	971,4	970,4	C+48	1.264,5	632,7	1.247,5	1.246,5	11
10	1.089,5	545,2	1.072,4	1.071,5	T	1.113,5	557,2	1.096,5	1.095,5	10
11	1.190,5	595,8	1.173,5	1.172,5	T	1.012,4	506,7	995,4	994,4	9
12	1.304,6	652,8	1.287,5	1.286,5	N	911,4	456,2	894,4		8
13	1.455,5	728,3	1.438,5	1.437,5	C+48	797,4	399,2	780,3		7
14	1.568,6	784,8	1.551,6	1.550,6	L	646,4	323,7	629,3		6
15	1.639,7	820,3	1.622,6	1.621,7	A	533,3		516,2		5
16	1.736,7	868,9	1.719,7	1.718,7	P	462,2		445,2		4
17	1.883,8	942,4	1.866,7	1.865,7	M+16	365,2		348,2		3
18	1.954,8	977,9	1.937,8	1.936,8	A	218,1		201,1		2
19	2.100,9	1.051,0	2.083,9	2.082,9	K	147,1		130,1		1

Sequence	Prob	SEQUEST XCorr	SEQUEST ΔCn	NTT	Modifications	Observed	Actual Mass	Charge	ΔDa	ΔPPM	Start	Stop	Spectrum ID
(K)HNIISNASC$_{153}$TTNC$_{157}$LAPMAK(V)	98%	4.3975	0.7408	2	Cys-SS-Cys (-2)	993,9636	1.985,91	2	-0,05367	-27,01	145	163	2min_NaoCl.03961.03961.2.dta
(K)HNIISNASC$_{153}$TTNC$_{157}$LAPMAK(V)	98%	4.6521	0.7062	2	Cys-SS-Cys (-2), Met-ox (+16)	1.001,96	2.001,91	2	-0,05298	-26,45	145	163	2min_NaoCl.03361.03361.2.dta
(K)HNIISNASC$_{153}$TTNC$_{157}$LAPMAK(V)	98%	2.0565	0.6463	2	Cys-SS-Cys (-2), Met-ox (+16)	1.001,97	2.001,92	2	-0,03687	-18,41	145	163	2min_NaoCl.03946.03946.2.dta

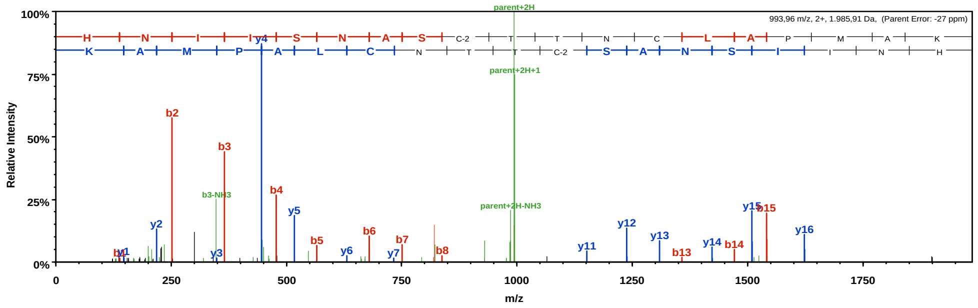

B	B Ions	B+2H	B-NH3	B-H2O	AA	Y Ions	Y+2H	Y-NH3	Y-H2O	Y
1	138,1	69,5			H	1.987,0	994,0	1.969,9	1.969,0	19
2	252,1	126,6	235,1		N	1.849,9	925,5	1.832,9	1.831,9	18
3	365,2	183,1	348,2		I	1.735,9	868,4	1.718,8	1.717,9	17
4	478,3	239,6	461,3		I	1.622,8	811,9	1.605,8	1.604,8	16
5	565,3	283,2	548,3	547,3	S	1.509,7	755,4	1.492,7	1.491,7	15
6	679,4	340,2	662,3	661,3	N	1.422,7	711,8	1.405,6	1.404,7	14
7	750,4	375,7	733,4	732,4	A	1.308,6	654,8	1.291,6	1.290,6	13
8	837,4	419,2	820,4	819,4	S	1.237,6	619,3	1.220,6	1.219,6	12
9	938,5	469,7	921,4	920,5	C-2	1.150,6	575,8	1.133,5	1.132,5	11
10	1.039,5	520,3	1.022,5	1.021,5	T	1.049,5	525,3	1.032,5	1.031,5	10
11	1.140,6	570,8	1.123,5	1.122,6	T	948,5	474,7	931,4	930,5	9
12	1.254,6	627,8	1.237,6	1.236,6	N	847,4	424,2	830,4		8
13	1.357,6	679,3	1.340,6	1.339,6	C	733,4	367,2	716,3		7
14	1.470,7	735,9	1.453,7	1.452,7	L	630,4	315,7	613,3		6
15	1.541,7	771,4	1.524,7	1.523,7	A	517,3		500,3		5
16	1.638,8	819,9	1.621,8	1.620,8	P	446,2		429,2		4
17	1.769,8	885,4	1.752,8	1.751,8	M	349,2		332,2		3
18	1.840,9	920,9	1.823,8	1.822,9	A	218,1		201,1		2
19	1.987,0	994,0	1.969,9	1.969,0	K	147,1		130,1		1

Figure S3C. LC-MS/MS fragment ion spectra for the detection of overoxidized Cys153-SO$_3$H and Cys157-SO3H peptides and the Cys153-SS-Cys157 intramolecular disulfide during GapDH inactivation by NaOCl in the absence of MSH. Gap was treated with 1 mM NaOCl for 2 min, followed by alkylation with IAM and tryptic digestion. The tryptic GapDH peptides were analyzed by Orbitrap mass spectrometry. The spectral counts, fragment ion spectra and tables show the formation of the several Cys153-SO$_3$H and Cys157-SO3H peptides as main thiol-oxidation form and less abundant intramolecular disulfides after treatment with NaOCl in vitro.

Figure S3D: Mycothiolation and intramolecular disulfide formation of GapDH of *C. diphtheriae* after treatment with 1 mM NaOCl

Sequence	Prob	SEQUEST XCo	SEQUEST ΔCr	NTT	Modifications	Observed	Actual Mass	Charge	ΔDa	ΔPPM	Start	Stop	Spectrum ID
(K)HNIISNASC$_{153}$TTNC$_{157}$LAPMAK(V)	99%	2.8589	0.6269	2	Carbamidomethyl (+57), ^ (+484), Met-ox (+16)	1.273,55	2.545,08	2	0,003081	1,21	145	163	2min_MSH_NaOCl.03296.03296.2.dta
(K)HNIISNASC$_{153}$TTNC$_{157}$LAPMAK(V)	87%	4.5166	0.648	2	Carbamidomethyl (+57), ^ (+484), Met-ox (+16)	849,3687	2.545,08	3	0,0038	1,492	145	163	2min_MSH_NaOCl.03286.03286.3.dta
(K)HNIISNASC$_{153}$TTNC$_{157}$LAPMAK(V)	87%	3.4862	0.5938	2	Carbamidomethyl (+57), ^ (+484), Met-ox (+16)	849,3683	2.545,08	3	0,002702	1,061	145	163	2min_MSH_NaOCl.03681.03681.3.dta
(K)HNIISNASC$_{153}$TTNC$_{157}$LAPMAK(V)	87%	3.4393	0.6977	2	Carbamidomethyl (+57), ^ (+484)	844,0364	2.529,09	3	0,001889	0,7465	145	163	2min_MSH_NaOCl.03709.03709.3.dta
(K)HNIISNASC$_{153}$TTNC$_{157}$LAPMAK(V)	87%	3.6355	0.6007	2	Sulfonic acid (+48), ^ (+484)	841,0237	2.520,05	3	0,000523	0,2074	145	163	2min_MSH_NaOCl.04261.04261.3.dta
(K)HNIISNASC$_{153}$TTNC$_{157}$LAPMAK(V)	87%	3.4735	0.584	2	^ (+484), ^ (+484)	986,4088	2.956,20	3	0,004036	1,365	145	163	2min_MSH_NaOCl.03649.03649.3.dta

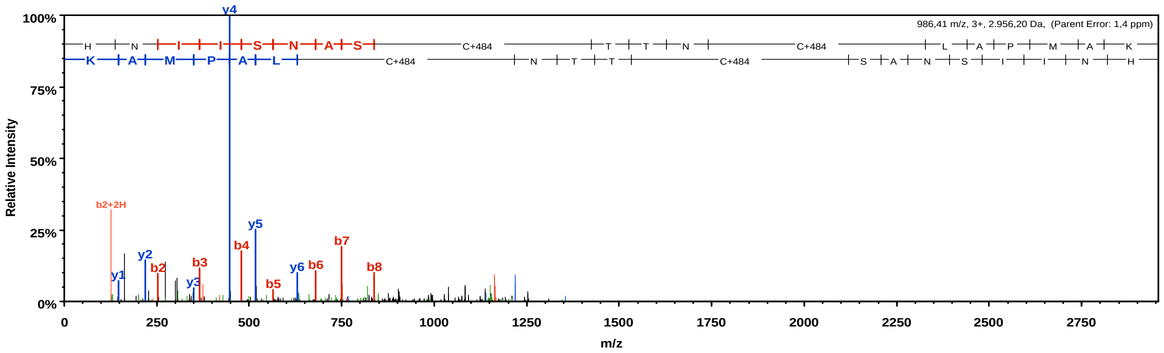

B	B Ions	B+2H	B-NH3	B-H2O	AA	Y Ions	Y+2H	Y-NH3	Y-H2O	Y
1	138,1	69,5			H	2.957,2	1.479,1	2.940,2	2.939,2	19
2	252,1	126,6	235,1		N	2.820,1	1.410,6	2.803,1	2.802,1	18
3	365,2	183,1	348,2		I	2.706,1	1.353,6	2.689,1	2.688,1	17
4	478,3	239,6	461,3		I	2.593,0	1.297,0	2.576,0	2.575,0	16
5	565,3	283,2	548,3	547,3	S	2.479,9	1.240,5	2.462,9	2.461,9	15
6	679,4	340,2	662,3	661,3	N	2.392,9	1.197,0	2.375,9	2.374,9	14
7	750,4	375,7	733,4	732,4	A	2.278,9	1.139,9	2.261,8	2.260,9	13
8	837,4	419,2	820,4	819,4	S	2.207,8	1.104,4	2.190,8	2.189,8	12
9	1.424,6	712,8	1.407,5	1.406,6	C+484	2.120,8	1.060,9	2.103,8	2.102,8	11
10	1.525,6	763,3	1.508,6	1.507,6	T	1.533,6	767,3	1.516,6	1.515,6	10
11	1.626,7	813,8	1.609,6	1.608,7	T	1.432,6	716,8	1.415,6	1.414,6	9
12	1.740,7	870,9	1.723,7	1.722,7	N	1.331,6	666,3	1.314,5		8
13	2.327,9	1.164,4	2.310,8	2.309,8	C+484	1.217,5	609,3	1.200,5		7
14	2.440,9	1.221,0	2.423,9	2.422,9	L	630,4	315,7	613,3		6
15	2.512,0	1.256,5	2.494,9	2.494,0	A	517,3	259,1	500,3		5
16	2.609,0	1.305,0	2.592,0	2.591,0	P	446,2	223,6	429,2		4
17	2.740,1	1.370,5	2.723,0	2.722,1	M	349,2	175,1	332,2		3
18	2.811,1	1.406,1	2.794,1	2.793,1	A	218,1	109,6	201,1		2
19	2.957,2	1.479,1	2.940,2	2.939,2	K	147,1	74,1	130,1		1

Sequence	Prob	SEQUEST XCo	SEQUEST ΔCr	NTT	Modifications	Observed	Actual Mass	Charge	ΔDa	ΔPPM	Start	Stop	Spectrum ID
(K)HNIISNASC$_{153}$TTNC$_{157}$LAPMAK(V)	99%	4.9592	0.7491	2	Cys-SS-Cys (-2), Met-ox (+16)	1.001,96	2.001,91	2	-0,05054	-25,23	145	163	2min_MSH_NaOCl.03444.03444.2.dta
(K)HNIISNASC$_{153}$TTNC$_{157}$LAPMAK(V)	99%	5.6037	0.6485	2	Cys-SS-Cys (-2)	993,9597	1.985,90	2	-0,0616	-31,01	145	163	2min_MSH_NaOCl.03975.03975.2.dta

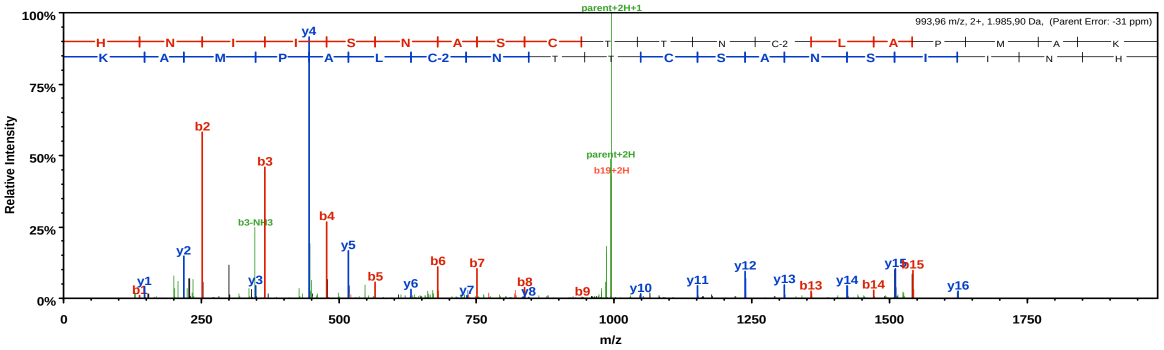

B	B Ions	B+2H	B-NH3	B-H2O	AA	Y Ions	Y+2H	Y-NH3	Y-H2O	Y
1	138,1	69,5			H	1.987,0	994,0	1.969,9	1.969,0	19
2	252,1	126,6	235,1		N	1.849,9	925,5	1.832,9	1.831,9	18
3	365,2	183,1	348,2		I	1.735,9	868,4	1.718,8	1.717,9	17
4	478,3	239,6	461,3		I	1.622,8	811,9	1.605,8	1.604,8	16
5	565,3	283,2	548,3	547,3	S	1.509,7	755,4	1.492,7	1.491,7	15
6	679,4	340,2	662,3	661,3	N	1.422,7	711,8	1.405,6	1.404,7	14
7	750,4	375,7	733,4	732,4	A	1.308,6	654,8	1.291,6	1.290,6	13
8	837,4	419,2	820,4	819,4	S	1.237,6	619,3	1.220,6	1.219,6	12
9	940,4	470,7	923,4	922,4	C	1.150,6	575,8	1.133,5	1.132,5	11
10	1.041,5	521,2	1.024,5	1.023,5	T	1.047,6	524,3	1.030,5	1.029,5	10
11	1.142,5	571,8	1.125,5	1.124,5	T	946,5	473,8	929,5	928,5	9
12	1.256,6	628,8	1.239,5	1.238,6	N	845,5	423,2	828,4		8
13	1.357,6	679,3	1.340,6	1.339,6	C-2	731,4	366,2	714,4		7
14	1.470,7	735,9	1.453,7	1.452,7	L	630,4	315,7	613,3		6
15	1.541,7	771,4	1.524,7	1.523,7	A	517,3		500,3		5
16	1.638,8	819,9	1.621,8	1.620,8	P	446,2		429,2		4
17	1.769,8	885,4	1.752,8	1.751,8	M	349,2		332,2		3
18	1.840,9	920,9	1.823,8	1.822,9	A	218,1		201,1		2
19	1.987,0	994,0	1.969,9	1.969,0	K	147,1		130,1		1

Figure S3D. LC-MS/MS fragment ion spectra for the detection of S-mycothiolated Cys153-SSM and Cys157-SSM peptides and the Cys153-SS-Cys157 intramolecular disulfide during GapDH inactivation by NaOCl in the presence of MSH. Gap was treated with 1 mM NaOCl for 2 min, followed by alkylation with IAM and tryptic digestion. The tryptic GapDH peptides were analyzed by Orbitrap mass spectrometry. The spectral counts, fragment ion spectra and tables show the formation of the several Cys153-SSM and Cys157-SSM peptides as main thiol-oxidation form and less abundant intramolecular disulfides after treatment with NaOCl in vitro.

Figure S4

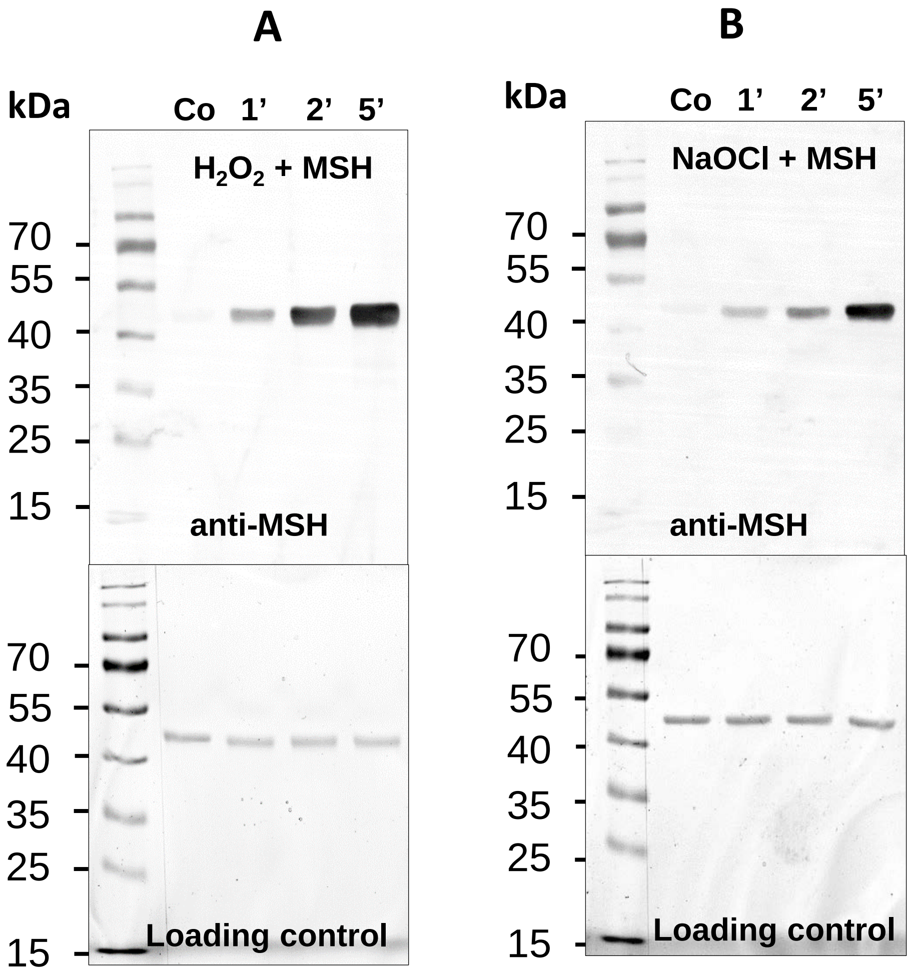

Figure S4. Gap is *S*-mycothiolated *in vitro* by H_2O_2 and NaOCl in the presence of MSH. MSH-specific Western blots confirmed the formation of the S-mycothiolated Gap under treatment with 1 mM H_2O_2 and MSH after 2-5 min **(A)** or with 1 mM NaOCl and MSH after 2-5 min **(B)** in the presence of MSH. The loading control is shown below the MSH-specific Western blot.
